# Longitudinal changes in moderate‐to‐vigorous‐intensity physical activity in children and adolescents: A systematic review and meta‐analysis

**DOI:** 10.1111/obr.12953

**Published:** 2019-10-23

**Authors:** Abdulaziz Farooq, Anne Martin, Xanne Janssen, Mathew G. Wilson, Ann‐Marie Gibson, Adrienne Hughes, John J. Reilly

**Affiliations:** ^1^ Research and Scientific Support Department Aspetar Orthopaedic and Sports Medicine Hospital Doha Qatar; ^2^ Social and Public Health Sciences Unit University of Glasgow Glasgow UK; ^3^ School of Psychological Science and Health University of Strathclyde Glasgow UK; ^4^ Institute of Sport, Exercise and Health University College London London UK

**Keywords:** accelerometer, adolescents, children, moderate‐to‐vigorous physical activity

## Abstract

Moderate‐to‐vigorous‐intensity physical activity (MVPA) is important for childhood obesity prevention and treatment, yet declines with age. Timing and magnitude of the decline in MVPA in children and adolescents are unclear but important for informing effective obesity intervention development. This systematic review aimed to determine and compare the year‐to‐year changes in MVPA among children and adolescents. Longitudinal studies were identified by searching 10 relevant databases up to December 2018. Studies were eligible for inclusion if they reported accelerometer‐assessed MVPA (min day^−1^) separately for boys and girls and had follow‐up duration of at least 1 year. After screening 9,232 studies, 52 were included representing 22,091 aged 3 to 18 year olds (boys=8,857; girls=13,234). Pooled‐analysis of the relative change in MVPA per year showed a decline of −3.4% (95% CI, −5.9 to −0.9) in boys and −5.3% (95% CI, −7.6 to −3.1) in girls, across all age groups. There were notable declines in MVPA at age 9 for both boys (−7.8%, 95% CI, −11.2 to −4.4) and girls (−10.2%, 95% CI, −14.2 to −6.3). The relative decline in MVPA affects both sexes from an early age; however, it is greater among girls. Interventions to promote MVPA should start before adolescence.

**Abbreviations**
MVPA: moderate‐to‐vigorous physical activity
ICAD: International Children's Accelerometry Database
RCT: randomized controlled trial
CI: confidence intervals
SMD: standardized mean difference
RE: random effects
QE: quality effects
CMA: comprehensive meta‐analysis
VPA: vigorous intensity physical activity
WHO: World Health Organization

## 1 INTRODUCTION

Moderate‐to‐vigorous intensity physical activity (MVPA) is associated with significant lifelong health benefits. Among adolescents for example, longitudinal studies have shown that higher MVPA is positively associated with favourable bone strength,^1^ cardio‐respiratory fitness,^2^, ^3^ blood pressure,^3^ lipid profile^4^ and insulin sensitivity.^5^ MVPA is particularly important in the prevention and treatment of child and adolescent obesity,^6^, ^7^ but levels are particularly low among children and adolescents with obesity^8^ and MVPA may have greater effects on body fatness amongst children and adolescents with higher levels of body fat.^6^ Among 7 year olds, a 10% increase in MVPA^9^ or 20‐minute increase in MVPA^10^ over 3 to 4 years significantly reduced body mass index *z* score. Current guidelines recommend that children engage in at least 60 minute day^−1^ of MVPA, yet many children and adolescents do not meet these recommendations globally.^11^, ^12^ Furthermore, based on few longitudinal studies with 5 years or more of follow‐up, MVPA appears to decline with age,^13^, ^14^, ^15^ but it is not clear how early in life this decline begins, or whether the rate of decline differs by sex. The Iowa Bone Development Study, which had the longest follow‐up of 12 years,^14^ found three distinct trajectories of activity from age 5 to age 17. In girls taking part in the Iowa Bone Development Study, absolute MVPA began to fall from age 5 years and had declined by 50 to 65% by age 17. In boys, absolute MVPA began to fall around age 8 years and had declined by 26 to 54% by age 17. The 10‐year cohort study (EarlyBird 41) found a linear decline in physical activity between age 9 and 15 in both sexes (total decline over that period compared with baseline was 20% in boys and 30% in girls).^15^ Contrary to these two longitudinal cohorts, the Gateshead Millennium Cohort study with 8‐year follow‐up found MVPA declined much earlier, from age 6 to 7 years in both sexes.^13^ Insights into the timing and magnitude of declines in MVPA could come from international consortium data such as the International Children's Accelerometry Database (ICAD), an important resource for accelerometer‐measured MVPA across childhood and adolescence; however, published analyses of the ICAD database is mostly cross sectional.^16^, ^17^ Understanding the timing and magnitude of MVPA decline among children and adolescents will be especially important to inform the design of targeted interventions to prevent MVPA decline and paediatric obesity.

Ideally, answering the questions of when and by how much MVPA declines with age would require large international longitudinal observational studies with extended follow‐up periods and quantitative (objective) measures of time spent engaging in MVPA. Unfortunately, few such global studies exist due to time and funding limitations.

Systematic reviews are needed to be confident about changes in MVPA across childhood and adolescence. The highly cited systematic review Dumith et al^18^ included 26 eligible studies, but 24/26 described changes in the total volume of physical activity rather than MVPA, the studies used subjective measures, were mostly from the United States and did not include children below 10 years of age. The use of questionnaires to obtain valid and reliable measures of MVPA has been generally unsuccessful and not recommended for younger children.^19^ With the advent of accelerometers, obtaining accurate estimates of MVPA^19^ has improved considerably across all age groups and especially in younger children. Moreover, in a systematic review, only studies that used device‐based measures of physical activity reported significant associations with overweight and obesity and were more likely to show associations with obesity prevalence.^20^ There is therefore a need for a new updated review and synthesis of the evidence in order to understand when and by how much objectively measured MVPA changes during childhood and adolescence. The aim of the present study was therefore to systematically review and critically appraise longitudinal studies that quantified year‐to‐year changes in accelerometer‐assessed MVPA among boys and girls 2 to 18 years of age.

### 1.1 METHODS

We used the 27‐item Preferred Reporting Items for Systematic Reviews and Meta‐Analyses (PRISMA) statement as a basis to report our systematic review. The systematic review protocol was registered at PROSPERO: CRD42015025036 (available from http://www.crd.york.ac.uk/PROSPERO/display_record.asp?ID=CRD42015025036).

### 1.2 Study eligibility

A study was eligible for inclusion if it met the following criteria: (a) randomized controlled trial (RCT) or longitudinal cohort studies of healthy children and adolescents 2 to 18 years of age and exposures reported separately for boys and girls at each age. For RCTs, only data from control subjects were considered if they did not receive any physical activity interventions; (b) MVPA was measured in minutes per day using accelerometers at least twice with at follow‐up of 1 year between measures; (c) the outcome of interest was annual percentage change in MVPA or absolute annual change in MVPA; and (d) published in English, due to resource constraints. These criteria were selected to minimize any seasonal influences on MVPA and because previous longitudinal studies have shown that the observed MVPA change from childhood to adolescence is generally not linear. Data collected at more than two time points were included in the systematic review and synthesis if the gap between each time point was 1 year. Studies were excluded if the participants had a disease or disability expected to impact MVPA. Studies where participants were part of physical activity interventions were excluded. Studies that used pedometers or mobile phone devices instead of accelerometers were not considered, since MVPA cannot be estimated accurately. For the meta‐analysis, in addition to the above criteria, studies with exactly 1 year of follow‐up were eligible to be able to quantify the annual MVPA change at each age.

### 1.3 Study selection and search strategy

Up to 31 December 2018, eligible studies were identified in the following databases: MEDLINE, Embase, PsycINFO, Scopus, PsycLit, Science Citation Index, SciTech Collection, Biological Sciences, Physical Education Index, and SPORTDiscus. The search strategy included medical subject headings and free‐text terms for the domains: study design, measurement tool, population and exposure. No publication date filters were applied. An example search strategy for the MEDLINE (OVID) platform is provided (Table S1).

Titles and abstracts were initially screened for eligibility by A. F.. The full‐text versions of potentially eligible studies were obtained and screened independently by two reviewers (A. F. and J. J. R.). Disagreements regarding study eligibility were resolved through discussion and/or additional information obtained from the study authors. For studies that did not report MVPA by age or for both sexes separately, corresponding authors were contacted by email to obtain this information. If no response was received after at least 3 reminders and 2 months or more, the study was deemed ineligible. Of the authors contacted, 32 of 42 eligible studies replied and shared the requested information. The response rate from corresponding authors was 76.2%. Of those authors who did not respond, 3 had changed institutions so no longer had access to the data and 7 were unable to respond due to their busy schedule.

### 1.4 Data extraction

An Excel spreadsheet was prepared to facilitate data extraction based on data items identified (Tables S2A and S2B). To reduce errors, duplicate data extraction was performed, in which AF extracted the data from all included studies on two occasions separated by ~3 months. All data extracted were checked and reviewed by two independent reviewers (A. M. G. and J. J. R.) for consistency and accuracy prior to data analysis.

From each paper, the following information was extracted and coded: (1) author and year of publication, (2) site/city/country where the study was performed, (3) year of baseline data collection, (4) length of follow‐up, (5) year of data collection at each follow‐up time point, (6) number of participants at baseline, (7) mean age of the participants at baseline, (8) number of participants at follow‐up, and (9) MVPA expressed as mean or median at baseline and at follow‐up time points.

In addition, the methods used for physical activity data collection using accelerometers were collected, based on Tanaka et al^21^: (a) brand of accelerometer used, (b) number of days accelerometer was worn, (c) minimum duration of wear time per day to be included in study, (d) minimum number of valid days of accelerometer data to be included in study, (e) whether accelerometer was worn on weekends, (f) the length of the recording interval duration used by the accelerometer to integrate data, and (g) cutoff points used to define MVPA.

### 1.5 Extraction of MVPA data

Often MVPA data for boys and girls were expressed as minute day^−1^ and was directly available from the studies at each age. In other studies, MVPA data were provided for population subgroups (e.g., by body mass index categories) or by weekend and weekday. In such cases, corresponding authors were contacted to provide MVPA data for each age and by sex. When data were not directly available, it was pooled using formulae and methods recommended in the Cochrane handbook for extracting data for continuous outcomes, as long as the sample size in each group and means were available.^22^ When MVPA data were subdivided into weekday and weekends, a 5:2 ratio weight was used to compute aggregate MVPA minute day^−1^ for the group. Two of the 52 included studies presented findings as figures with MVPA on the *y*‐axis. Here data were extracted using Plot digitizer software, which has a reliability of up to 93% for a precision of 0.1 unit.^23^

### 1.6 Risk of bias assessment

The Cochrane Collaboration tool for assessing risk of bias in longitudinal observational studies or RCTs was used that includes five domains of bias (selection, performance, attrition, reporting, and other)^21^, ^24^ (Table S3). Author A. F. paired with A. M., A. H., or X. J. to appraise the included studies for the presence of risk of bias and judged the level of bias for each of the five domains (high bias, low bias or unclear bias). Any disagreement in a domain was resolved by discussion to reach a consensus rating.

### 1.7 Summary measures

The primary summary measure for this systematic review was the annual relative change in participant's accelerometer‐assessed MVPA in minute day^−1^. Participants' MVPA at baseline and at subsequent follow‐up of one or more years was recorded. A variety of accelerometry cut‐off points have been used to define MVPA^25^; we did not exclude studies based on the particular cut‐off point used to define MVPA. To synthesize data, we calculated relative change in MVPA as the percentage change from the baseline value (calculated as [follow‐up MVPA minus baseline MVPA]/baseline MVPA × 100). When there was a follow‐up duration of more than 1 year, relative change was divided by the number of follow‐up years, as described previously.^18^

### 1.8 Data analysis

Data were presented as frequencies and percentages or means with 95% confidence intervals (CIs) as appropriate. The extracted data on MVPA (min day^−1^) from all included studies were plotted against age on the *x*‐axis to display trajectories separately for boys and girls. To determine if annual percentage change in MVPA was significantly different from zero, a one‐sample paired *t* test was performed for each variable category.

To estimate the pooled annual change in MVPA (min day^−1^), mean change in MVPA at 1‐year follow‐up from baseline and standard deviation of the change were combined in a meta‐analysis using random effect (RE) models. Missing standard deviations of the change in MVPA from baseline were calculated from *P*‐values, CIs or standard errors, if available.^22^ Alternatively, the standard deviation of the change was computed when correlation coefficients between baseline and follow‐up measurements were available and vice‐versa.^22^ Where it was not possible to calculate the standard deviation for the change (n=13 studies), hypothesized correlation coefficients of *r*=0.3, *r*=0.5 and *r*=0.7 were used to compute the effect sizes.^22^ A sensitivity analysis was conducted to assess the robustness of the pooled effect sizes for the three hypothesized correlation coefficients (*r*=0.3, *r*=0.5 and *r*=0.7). Based on available data, the mean correlation coefficient was *r*=0.51; therefore, computed effects sizes presented in this paper are based on a correlation coefficient *r*=0.5. We conducted a sensitivity analysis based on recommendations from Doi et al,^26^ using a quality effects (QE) model of meta‐analysis that assigns more weight to larger studies and studies with higher scores on quality assessment.

Certain issues are common in single‐group pre‐post studies for meta‐analyses^22^, ^27^ such as the baseline characteristics of the study populations are often not similar across studies; the magnitude of change in outcome measure (whether a decline or increase) often depends on the baseline value, and with studies involving longer follow‐up durations, the attrition rates may be higher, thereby contributing to bias in the estimates due to imbalance in cofounding factors. To address these issues, we controlled for age and sex at baseline across all studies in the meta‐analysis using subgroups and included only those studies that represent a 1‐year change (n=31). To allow interstudy comparisons, we generated standardized paired mean difference (SMD) as a measure of effect. The SMD expresses the effect size relative to the variability observed^22^; hence, studies will have same SMD, where the difference in means is the same proportion of the SD. Effect sizes (SMD) were considered as small, medium, and large for 0.2, 0.5, and 0.8, respectively.^28^ Meta‐analysis was performed separately for boys and girls using RE models presented as forest plots. Heterogeneity across studies was assessed using *I* ^2^ statistics (*I* ^2^ of 0‐40% represents low heterogeneity and 75‐100% considerable heterogeneity).^22^ Bubble plots were presented to display the association of MVPA declines with age and sex. Publication bias was estimated using funnel plots (a scatter plot of effect sizes and standard errors from each study) and asymmetry was diagnosed based on visual inspection. Publication bias was considered when *P*‐value was <.05 using Egger's test.^29^ Statistical software, STATA ver 15.0 (STATA Corp., Texas, USA), was used for statistical analysis, while Comprehensive Meta‐Analysis ver 3.3 (Biostat, Englewood, NJ,USA) for running the meta‐analysis.

## 2 RESULTS

### 2.1 Study selection

**Figure 1:**
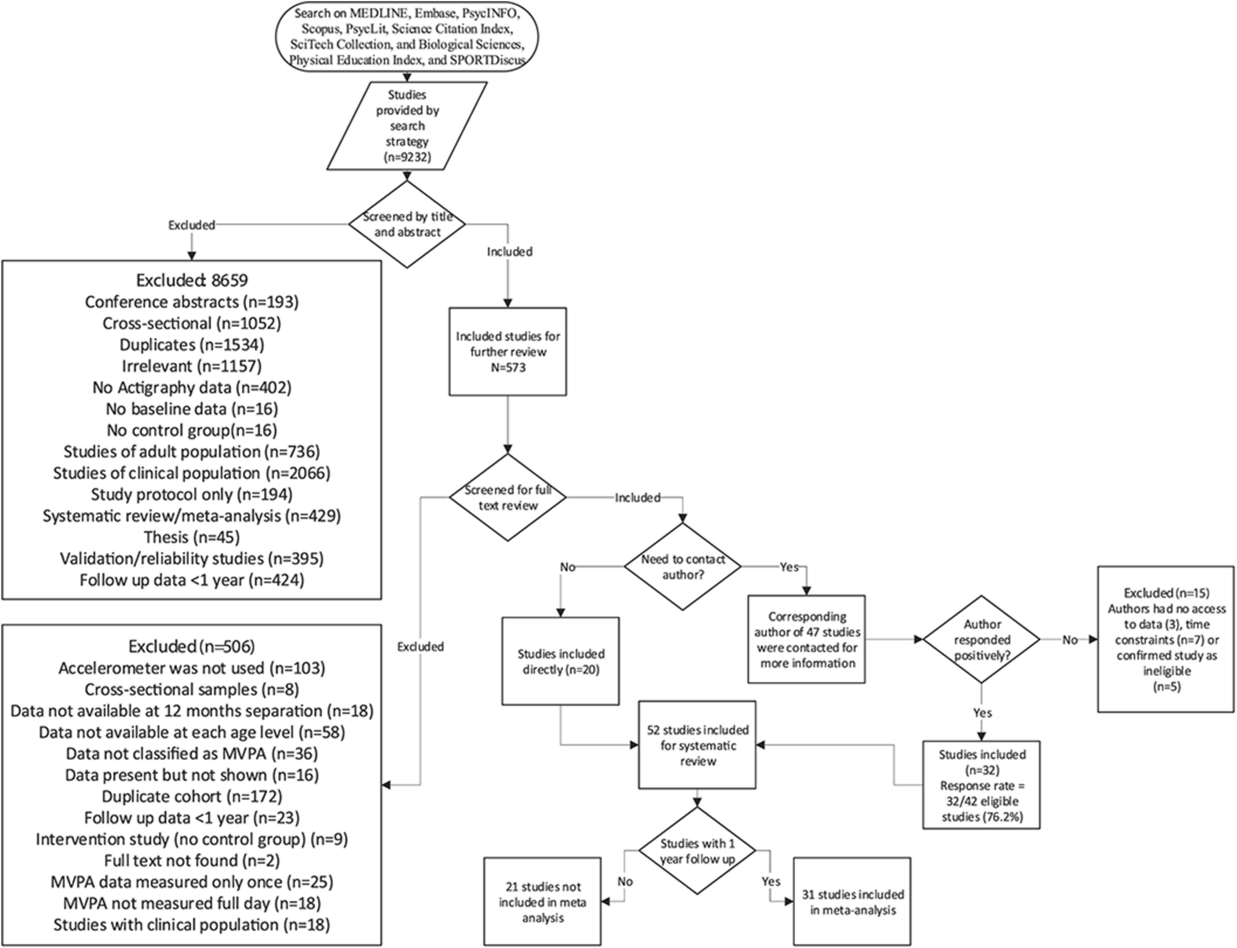
Flow chart through different phases of systematic review (identification, screening, eligibility, inclusion)

The initial search identified 9,232 records. The title and abstract of unique records (7,125) were screened. Among them, 573 studies were eligible for an in‐depth review of their full text. Based on the review inclusion criteria and data availability, 521 studies were further excluded. Thus, 52 studies^13^, ^14^, ^16^, ^30^, ^31^, ^32^, ^33^, ^34^, ^35^, ^36^, ^37^, ^38^, ^39^, ^40^, ^41^, ^42^, ^43^, ^44^, ^45^, ^46^, ^47^, ^48^, ^49^, ^50^, ^51^, ^52^, ^53^, ^54^, ^55^, ^56^, ^57^, ^58^, ^59^, ^60^, ^61^, ^62^, ^63^, ^64^, ^65^, ^66^, ^67^, ^68^, ^69^, ^70^, ^71^, ^72^, ^73^, ^74^, ^75^, ^76^, ^77^, ^78^, ^79^ met the inclusion criteria and were included in the quantitative analysis representing a total sample of 22,091 children and adolescents (Figure 1; PRISMA flow diagram). A subset of 31 studies with exactly 1 year of follow‐up data were included in the meta‐analysis.

### 2.2 Study characteristics

Table 1 shows the characteristics of all studies included in the systematic review. Studies with girls were overrepresented and a total of 13,234 girls and 8,857 boys provided data for the subsequent analysis. The data were mostly from children living in high‐income regions (99.1% of the study participants).

**Table 1:** Characteristics of the included studies (n=52) in the systematic review

Overall	Cohorts^a^	Sample Size at Baseline
All 52 studies	124	22,091
Sex		
Boys	58	8,857
Girls	66	13,234
Region		
Australia/New Zealand	27	2,302
Europe	51	4,712
Mexico	2	205
United Kingdom	27	10,261
United States	21	4,611

a Some studies will have more than one cohort representing children at different age, sex and time points.

In at least half of the eligible studies, participating children and adolescents wore the accelerometer for at least 7 days. Almost all studies included weekend days. The definition of MVPA thresholds varied across studies, but the majority of studies used the Actigraph and Evenson's cut‐off of 2,296 counts per minute to define MVPA. In 29.4% of included studies, the Epoch length was 60s. For the accelerometer data to be considered valid, almost all cohorts required 8 hours or more of data each day and at least 3 days of valid accelerometer data (Table 2).

**Table 2:** Methods used to assess, validate and define MVPA using accelerometers in the included studies

	N	Percent
Brand of accelerometer		
Actical	2	3.9
Actigraph	42	82.4
Actiheart	2	3.9
Sensewear	2	3.9
RT3	2	3.9
GENEActiv	1	2.0
No. of wear days		
3	4	7.8
4	7	13.7
5	8	15.7
6	3	5.9
7	25	49.0
8	3	5.9
Not mentioned	1	2.0
Included weekend		
Yes	46	90.2
No	5	9.8
Epoch length		
≤15	19	37.3
30	8	15.7
60	15	29.4
Not mentioned	9	17.3
Wear time for valid day		
6‐9 h	23	44.2
10 h	18	34.6
12‐16.6	5	9.6
Not mentioned	6	11.5
Valid day		
1	4	7.8
2	7	13.7
3	26	51.0
4	6	11.8
5	1	2.0
6	1	2.0
Not mentioned	6	11.8
Definition of MVPA		
≥4 METS	8	15.7
816 to 1,500 cpm	5	9.8
2,000 cpm	7	13.7
2,296 cpm	22	43.1
≥3,000 cpm	3	5.9
Freedson	2	3.9
Other	2	3.9
Not mentioned	2	3.9

Abbreviation: MVPA: moderate‐to‐vigorous‐intensity physical activity.

### 2.3 Risk of bias within studies

In the risk of bias assessment, around one fifth of the included studies were judged as high risk of bias, because of lack of random selection and convenient sampling in the population recruited (Table S3). Performance bias was rated as unclear (24%) or high (20%) when studies used 2 days or 1 day of valid wear time (>8 h), respectively, to estimate MVPA. The highest bias was reported in the attrition domain (55%). Around 33% confirmed that characteristics of the participants with missing and without missing MVPA data were similar at baseline. There were no studies with selective reporting bias or other sources of bias due to the strict inclusion criteria of our study.

### 2.4 Mean MVPA change per year

**Figure 2:**
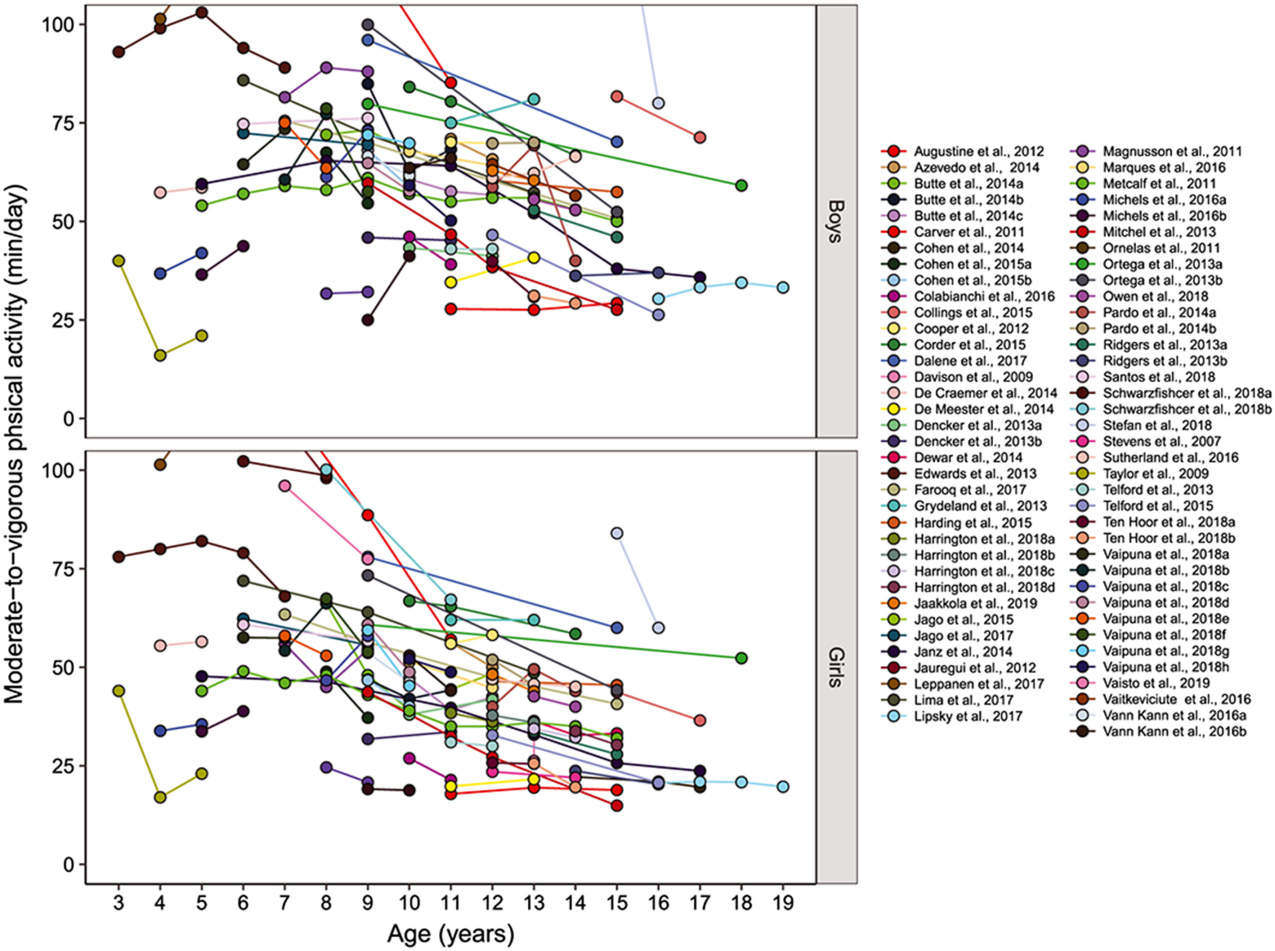
Trajectories of moderate‐to‐vigorous‐intensity physical activity among boys and girls from age 3 to age 18 years

The data extracted on average time spent in MVPA at baseline and at each consecutive follow‐up measurement is presented in Figure 2. Mean MVPA is often below the 60 minute day^−1^ recommendation, and it declines across most of the paediatric age range. There was considerable variation between studies in the magnitude of the decline, in part related to methodological differences and in part due to real differences between samples and settings: mean duration of annual change in daily MVPA ranged from −63.0 to 20.7 minutes with an average of −3.4 minutes, 95% CI (−4.6 to −2.2) and a SD of ±8.8 minutes.

### 2.5 Mean percentage MVPA change per year

Based on MVPA data from all 52 included studies (Figure 2), the percentage MVPA change per year was determined and is presented in Table 3. Girls showed greater % decline per year in MVPA (−5.3%; 95% CI, −7.6 to −3.0) than boys (−3.5%, 95% CI, −6.0 to −0.9). Although the percentage MVPA change per year was always negative from age 6 among girls and from age 8 among boys, significant declines were observed in girls at ages 6 years (−6.4%, 95% CI, −10.0 to −2.7), 9 years (−10.2%, 95% CI, −14.2 to −6.3), 10 years (−7.9%, 95% CI, −15.0 to −0.7), and 13 years (−8.5%, 95% CI, −12.8 to −4.3). As for the boys, percentage MVPA change per year was statistically significant at age 9 years (−7.8%, 95% CI, −11.2 to −4.4). Combining data of boys and girls, the annual declines in MVPA peaked twice across the entire age range; once at age 9 years (−9.0%, 95% CI, −11.5 to −6.6) and then at 13 years (−8.4%, 95% CI, −13.2 to −3.7). The percentage MVPA change per year was higher and statistically significant on weekends (−5.3%, 95% CI, −9.5 to −1.0) compared with weekdays (−3.1%, 95% CI, −7.5 to 1.3) based on available weekday‐weekend data from 13 of 52 studies.

**Table 3:** Average annual percentage change in MVPA (%) in boys and girls and by age groups and day of the week (n = 52 studies)

	Boys			Girls		
	Annual Percentage Change, %	95% Confidence Interval	*P*‐Value	Annual Percentage Change, %	95% Confidence Interval	*P*‐Value
Overall	−3.46	−6.02 to −0.90	.008	−5.28	−7.59 to −2.97	<.001
Age						
3	−26.8	−448.9 to 395.4	.568	−29.4	−435.5 to 376.7	.527
4	13.3	−1.1 to 27.6	.062	11.8	−5.7 to 29.3	.134
5	5.0	−13.6 to 23.6	.457	5.5	−9.2 to 20.1	.320
6	0.3	−5.4 to 5.9	.920	−6.4	−10.0 to −2.7	.004
7	2.1	−13.2 to 17.5	.736	−3.2	−18.6 to 12.3	.622
8	−3.8	−15.8 to 8.2	.479	−6.5	−22.9 to 9.8	.375
9	−7.8	−11.2 to −4.4	<.001	−10.2	−14.2 to −6.3	<.001
10	−3.8	−9.8 to 2.2	.186	−7.9	−15.0 to −0.7	.035
11	−2.2	−7.2 to 2.8	.349	−1.3	−6.0 to 3.3	.540
12	−4.4	−11.0 to 2.2	.168	−3.0	−9.3 to 3.3	.314
13	−8.4	−19.1 to 2.4	.112	−8.5	−12.8 to −4.3	.001
14	−2.3	−45.3 to 40.7	.623	−5.5	−11.4 to 0.5	.065
15	−17.1	−75.2 to 41.0	.334	−12.7	−47.0 to 21.7	.253
16	−			−2.7	−53.1 to 47.7	.619
Day of the week^a^						
On weekdays	−3.4	−10.8 to 4.1	.346	−3.0	−9.7 to 3.7	−9.7 to 3.7
On weekends	−5.3	−14.0 to 3.3	.203	−4.3	−9.9 to 1.3	−9.9 to 1.3

Abbreviation: MVPA: moderate‐to‐vigorous‐intensity physical activity.

a n=13 studies.

### 2.6 Meta‐analysis of annual change in MVPA minutes (paired mean difference)

**Figure 3:**
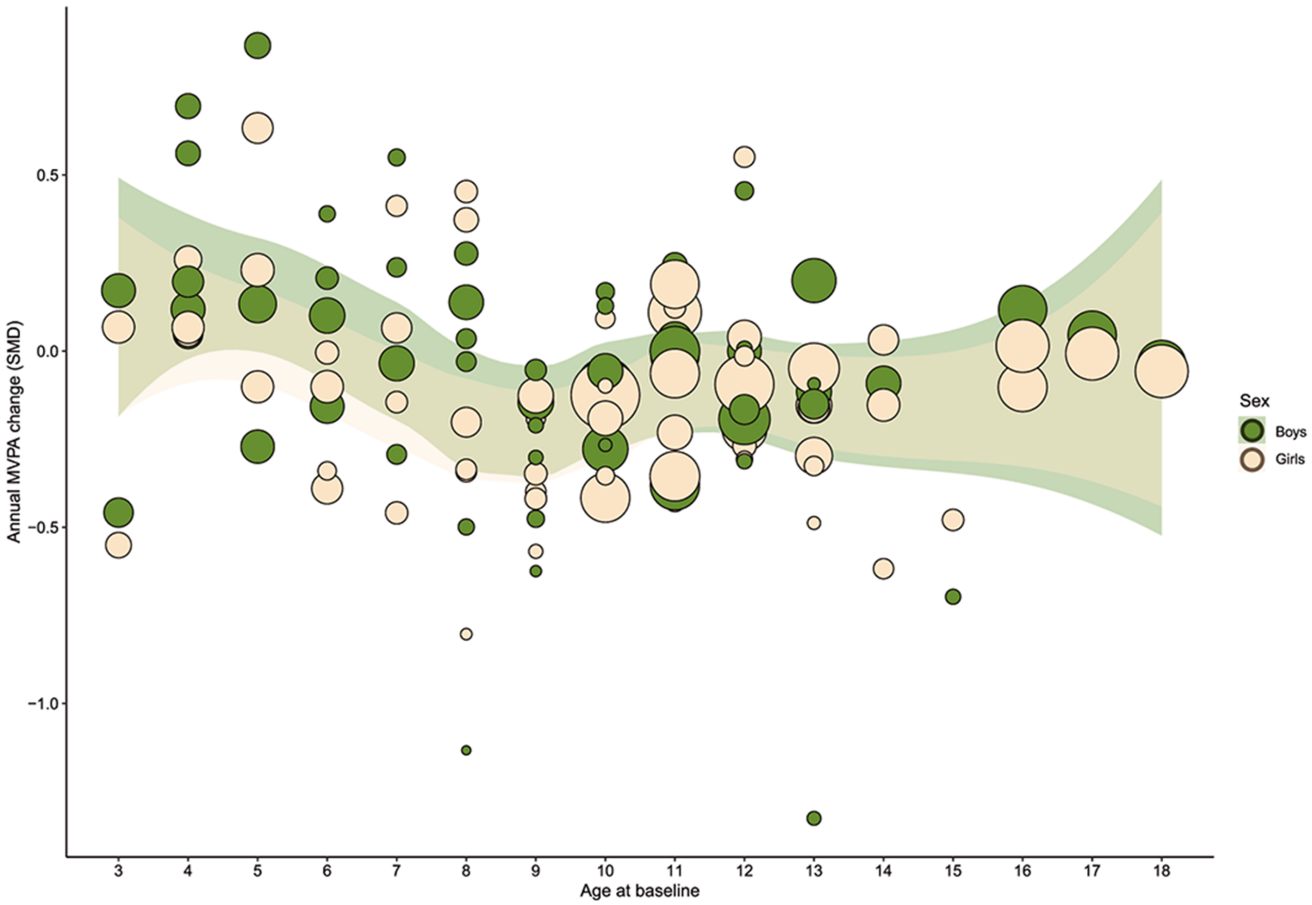
Scatter plot of standardized mean annual difference in moderate‐to‐vigorous‐intensity physical activity against age at baseline in boys and girls **Legend:** The bubbles represent the estimates of effect sizes using random effects derived from meta‐analysis with covariates, age at baseline, and gender. Size of bubbles are proportionate to the level of precision of each effect in the study; the projected 95% CI shaded region is a loess function curves for boys and girls respectively

A subset of 31 studies with exactly 1 year of follow‐up data were included in the REs meta‐analysis to quantify the annual change in MVPA at each age (Figure 3). The bubbles represent SMD of annual MVPA change. Larger bubbles show that studies with more power and bubbles below zero indicate declines. While there was clear evidence of declining MVPA across childhood and adolescence from an early age, the decline was generally nonlinear in both boys and girls (Figure 3).

**Figure 4:**
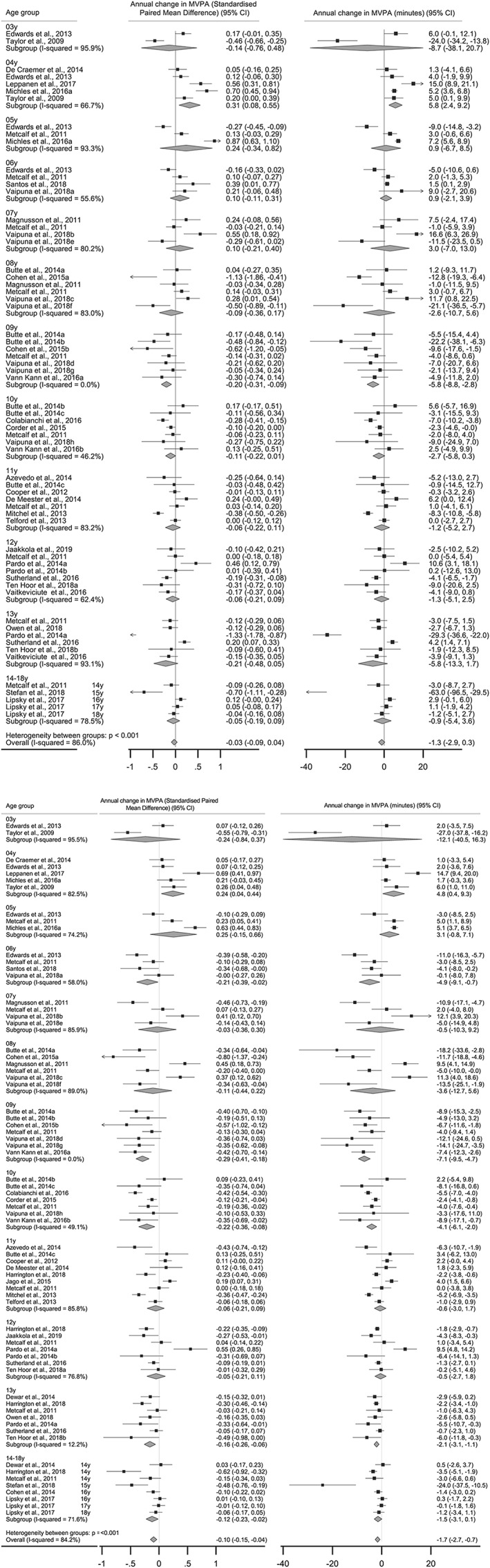
A, Random effects meta‐analysis of the annual mean difference in moderate‐to‐vigorous physical activity (min day^−1^) based on age level among boys. B, Random effects meta‐analysis of the annual mean difference in moderate‐to‐vigorous physical activity (min day^−1^) based on age level among girls

The forest plots of the RE model for boys (Figure 4A) and girls (Figure 4B) provides both actual changes in minutes of MVPA and SMD separately (n=31 studies). Combined annual change in MVPA across all ages, expressed as the paired SMD, was −0.03 (95% CI, −0.09 to 0.04) among boys and −0.10 (95% CI, −0.15 to −0.04) among girls. Age‐specific subgroup analysis indicated a statistically significant decline in MVPA among boys age 9 (SMD: −0.20, 95% CI, −0.31 to −0.09). Among girls, the meta‐analysis (Figure 4B) revealed multiple ages at which MVPA declined significantly starting as early as ages 6, 9, 10 and again at age 13 corresponding to reduction of MVPA SMDs: −0.21, 95% CI (−0.39 to −0.02); −0.29, 95% CI (−0.41 to −0.18); −0.22, 95% CI (−0.36 to −0.08), and −0.16, 95% CI (−0.26 to −0.06) respectively.

In terms of heterogeneity, meta‐analysis provided a heterogeneity statistics *I* ^2^ of 86.0% for boys and *I* ^2^ of 84.2% for girls. This indicated substantial inconsistency across studies and reflective of different differences found at each age. Funnel plots in Figure S1 show acceptable levels of symmetry suggesting the absence of publication bias. This was confirmed by Egger's tests that provided nonsignificant values for effect sizes representing studies from boys (*P*=.921) and girls (*P*=.455).

### 2.7 Sensitivity analysis

In the sensitivity analysis, pooled effects of annual MVPA change were similar to effects obtained if the correlation coefficients for imputing missing SDs were changed to *r*=0.3 or *r*=0.7 (Table S4). Decline in MVPA among boys age 10 years was statistically significant when considering the QE model that gives more weight to higher quality studies (Table S5).

## 3 DISCUSSION

This review found a large number of studies on objectively measured longitudinal changes in MVPA across childhood to adolescence. The key findings are that there is a significant annual decline in MVPA across all age groups from around age 6 years onwards in girls and from age 9 among boys. Although statistically significant, these annual changes were mostly of moderate effect sizes with SMD just above 0.2 in most cases. The notable annual declines in MVPA at age 9, for boys (−7.8%) and (−10.2%) in girls are roughly equivalent to a 6 minute per day decline in MVPA per year. Annual declines during adolescence were similar to annual declines during childhood. Systematic review findings from this study have important implications, since the amount of time per day spent in MVPA is important to the prevention and treatment of paediatric obesity^6^, ^20^ and seems to be particularly important as an influence on body fatness in those children and adolescents who are living with obesity.^6^ In summary, practitioners, policymakers, and parents should consider that in the absence of interventions, MVPA will decline year on year well before adolescence.

Investigators in individual studies have found varying timing and magnitudes of declines in MVPA and have usually compared their findings against a small number of other similar studies.^77^, ^79^, ^80^ In general, there has often been a lack of clarity in previous individual studies regarding the timing and magnitude of age‐related changes in MVPA across childhood and adolescence. The systematic review process has enabled a more comprehensive assessment of the available evidence than was possible in individual studies and has provided some clarity as to when and by how much MVPA changes with age. The results from this systematic review, however, provide information about expected average change in a group and do not reflect individual trajectories. There is an emerging body of evidence of individual differences in the timing and magnitude of declining MVPA with age in children and adolescents.^13^, ^81^ For example, Kwon et al found four distinct trajectories and showed that active children who become less active with age are more likely to live with obesity in young adulthood.^82^

Previously, Dumith et al^18^ described physical activity change throughout adolescence and observed that the decline in physical activity was fairly consistent across 10 to 18 years. Since Dumith et al^18^ was mostly based on studies of questionnaire‐assessed physical activity, did not focus on MVPA, and did not include children less than 10 years of age, this older review is not directly comparable with the present review. The ICAD has pooled accelerometry data from across childhood and adolescence, mostly from high‐income countries, and has reported cross‐sectional differences (apparent declines) in MVPA across the entire period of childhood and adolescence from around age 5 years. The findings of the ICAD data analyses are consistent with the findings of the present review in that they have reported that MVPA decline begins in early‐mid childhood, occurs in both sexes and is not especially marked around adolescence.^17^

A recent longitudinal study from the United Kingdom across the age range 6 to 15 years found that objectively measured sedentary time increased across the age range, displacing both MVPA and light intensity physical activity over time.^83^ In the present review, the percentage MVPA change per year observed at age 9 and age 13 in boys and girls is consistent with physical activity declines from the ICAD pooled cross‐sectional studies at ages 9 to 10 and ages 12 to 13.^16^

Also, previous studies^39^ have suggested that the annual decline in MVPA is greater on weekends compared with weekdays, supported by our analysis of a subset of 13 studies in which the age‐related decline in MVPA on weekends was higher than on weekdays.

### 3.1 Study and evidence‐base strengths and limitations

The main strength of this study is that despite the strict inclusion criteria to only include accelerometer‐measured and longitudinal MVPA data, a relatively large number of studies were identified and included. We contacted authors to provide the data in the format that answered our specific research question with a response rate of 76.2%. This adds credibility to the results. The study also represents an advance on the previous systematic review of the topic^18^ by extending the age range across childhood and adolescence, including objectively measured change data only, and the focus on MVPA, the most health enhancing intensity of physical activity, and the component of physical activity which is of most importance to the development and maintenance of paediatric obesity.

The present study however had some limitations. The literature search was limited to studies written in English and from countries where accelerometers were readily available. Therefore, the review primarily represents evidence largely from high‐income countries. Secondly, while accelerometers provide valid and acceptable measures of MVPA, they can underestimate MVPA in certain situations e.g. cycling or swimming.^84^ A weakness often not considered was some of the included studies in the present review represented control group data from RCTs, often with different research objectives to those of the present study. A subgroup analysis was performed to compare if the estimates obtained from RCTs were similar to effects obtained from other study designs and whether employing a different epoch length during accelerometer assessment influenced the parameters estimates. The results across study designs were similar, but epoch length categories revealed different estimates of the annual MVPA decline. Shorter epochs (<15s) were used in studies involving younger children, and this could explain the smaller estimates of change in MVPA per year when compared with longer epochs (60s) used in the older age groups. (Table S6).

There was attrition due to invalid accelerometer data at follow‐up in most studies. Among the eligible studies 55% did not report whether characteristics of the participants lost to follow‐up were similar to those retained. This was not ideal but may have had relatively little impact on our results because in most cases, the numbers of individuals lost to follow‐up were minimal and much of the invalid accelerometer data can possibly be assumed as missing at random. We specifically also asked the corresponding authors to provide valid MVPA change data at every wave of data collection, instead of data for the largest sample at each wave. Moreover, by performing the QE model that gives less weight to studies with high risk of bias with respect to selection, performance, detection, and attrition domain (studies with lower overall quality score), observed effect sizes did not change significantly. (Table S5). However, as per QE model, age 10 boys also showed significant annual decline in MVPA.

We also acknowledge the inability to gather data on explanatory or confounding variables, which may influence changes in MVPA across childhood and adolescence such as adiposity, pubertal maturation, socio‐economic status and other potential predictors like season, weekday/weekend. In most cases, the explanatory and confounding factors were not readily available from the published studies, and if we had requested this information, the response rate from the corresponding authors could have been reduced due to the complexity of the request. By focusing on key variables associated with change (age, sex and MVPA), we were able to address their possible influence on age‐related declines in MVPA, but the influence of other variables remains to be investigated in future research.

Vigorous intensity physical activity (VPA), as distinct from MVPA, may be particularly important in the development and maintenance of child and adolescent obesity,^91,96^ and VPA also seems to decline with age from early‐mid childhood.^85^ However, most of the studies included in this review did not provide VPA separately and so age‐related declines in VPA could not be considered. Alternative approaches to understanding age‐related declines in MVPA or VPA after controlling for seasonality, weekday/weekend, or other factors might be possible utilizing the data from the ICAD or Biobanks.

Our review includes studies across a wide age range from 2 to 18 years, but there were fewer studies of changes in MVPA from early childhood than later in childhood, and far fewer studies of changes in MVPA in late adolescence, above 15 years old (Figure 2). This observation highlights the need for more longitudinal studies in these age groups. Relatively low numbers of studies in these age groups could be due to the common belief among researchers that MVPA changes occur only around adolescence and the erroneous belief that MVPA in early childhood and/or late adolescence is unimportant.^86^ Research experts in the field of physical activity must overcome the common challenges of working with younger children and older adolescents such as recruitment, compliance with accelerometry and participant retention in order to address these research gaps.

The accelerometry data reduction decisions made in individual studies, such as thresholds to define MVPA, varied by study. Our decision to compute the percentage decline in MVPA per year as done in a previous review^18^ allowed for a comparison of change in time across studies despite these differences in methodology.

### 3.2 Study implications

The present review suggests that MVPA decline begins in early to midchildhood and occurs in both sexes; however, the rate of decline rate was slightly greater in girls than boys. Future policymaking and practice in paediatric obesity prevention and treatment might usefully attempt to promote MVPA during childhood and continue through adolescence. The absence of statistically significant annual declines between the ages of 16 to 18 years in this review does not imply that physical activity interventions are not needed. A potential reason for nonsignificant findings is that MVPA levels were already very low in these age categories. In summary, two lines of evidence emphasize the urgent need for interventions, which attempts to maintain MVPA/prevent the age related decline in MVPA.^87^, ^88^ First, the widespread evidence globally that many children and adolescents are not meeting the recommended 60 min day^−1^ MVPA. Second, the clear evidence from the present study that there is a decline in MVPA each year from early childhood.

## 4 CONCLUSIONS

Habitual time spent in MVPA is critical to child and adolescent obesity prevention and treatment according to evidence based guidelines. Future research, policy or practice interventions aimed at preventing age‐related declines in MVPA need to begin earlier in childhood, continue through adolescence and include both boys and girls.

## ETHICS APPROVAL AND CONSENT TO PARTICIPATE

Not applicable to systematic reviews and meta‐analysis.

## CONSENT FOR PUBLICATION

Not applicable.

## AVAILABILITY OF DATA AND MATERIAL

The data used in the current study can be extracted from Tables S2A and S2B or are available from corresponding authors on request.

## COMPETING INTERESTS

All the authors declare that they have no competing interests.

## FUNDING

A. M. was supported by the UK Medical Research Council (grant number MC_UU_12017/14) and the Scottish Government Chief Scientist Office (grant number SPHSU14).

## AUTHORS' CONTRIBUTIONS

AF and JR formulated and conceived the research question. AF performed the evidence search and screened the studies for eligibility. AF and JR conducted full text screening. AF, AM, XJ and AH did the risk of bias assessment for each study. Data extraction and inspection was collectively performed by AF, JR and AMG. AF performed the data analysis. All authors did the data interpretation, reviewed and approved the final manuscript.

## Supporting information

Table S1. Sample search strategy using OVID
Table S2A. Description of included studies and methods used for objective physical activity assessment
Table S2B. Moderate‐to‐vigorous physical activity (min/day) at baseline and subsequent follow up each year among boys and girls in the included studies
Table S2B. Moderate‐to‐vigorous physical activity (min/day) at baseline and subsequent follow up each year among boys and girls in the included studies
Table S3: Risk of Bias assessment and summary
Figure S1. Contour enhanced funnel plot for random effects meta‐analysis of standardised mean MVPA difference per year in Boys and girls
**Table S4. Sensitivity analysis showing estimated annual MVPA change (paired standardised mean difference) according to age in boys and girls assuming correlation coefficients for 0.3, 0.5 and 0.7.**
**Table S5. Sensitivity analysis showing estimated annual MVPA change (paired standardised mean difference) according to age in boys and girls using random effects (RE) model and quality effects (QE) model**
**Table S6. Sensitivity analysis showing estimated annual MVPA change (paired standardised mean difference) according to age in boys and girls using random effects (RE) model and quality effects (QE) model**

## References

[1] Janz KF , Letuchy EM , Burns TL , Gilmore JME , Torner JC , Levy SM . Objectively measured physical activity trajectories predict adolescent bone strength: Iowa Bone Development Study. Br J Sports Med. 2014;48(13):1032‐1036. 24837241 10.1136/bjsports-2014-093574 PMC4550443

[2] Janz KF , Dawson JD , Mahoney LT . Tracking physical fitness and physical activity from childhood to adolescence: the muscatine study. Med Sci Sports Exerc. 2000;32(7):1250‐1257. 10912890 10.1097/00005768-200007000-00011

[3] Carson V , Rinaldi RL , Torrance B , et al. Vigorous physical activity and longitudinal associations with cardiometabolic risk factors in youth. Int J Obes (Lond) (*2005* 2014;38(1):16‐21. 23887061 10.1038/ijo.2013.135

[4] Raitakari OT , Taimela S , Porkka KV , et al. Associations between physical activity and risk factors for coronary heart disease: the Cardiovascular Risk in Young Finns Study. Med Sci Sports Exerc. 1997;29(8):1055‐1061. 9268963 10.1097/00005768-199708000-00011

[5] Henderson M , Benedetti A , Gray‐Donald K . Dietary composition and its associations with insulin sensitivity and insulin secretion in youth. Br J Nutr. 2014;111(3):527‐534. 24047611 10.1017/S0007114513002572

[6] Janssen X , Basterfield L , Parkinson KN , et al. Non‐linear longitudinal associations between moderate‐to‐vigorous physical activity and adiposity across the adiposity distribution during childhood and adolescence: Gateshead Millennium Study. Int J Obes (Lond). 2019;43(4):744‐750. 30108270 10.1038/s41366-018-0188-9 PMC6484716

[7] Mann KD , Howe LD , Basterfield L , et al. Longitudinal study of the associations between change in sedentary behavior and change in adiposity during childhood and adolescence: Gateshead Millennium Study. Int J Obes (Lond). 2017;41(7):1042‐1047. 28293017 10.1038/ijo.2017.69 PMC5500163

[8] Elmesmari R , Martin A , Reilly JJ , Paton JY . Comparison of accelerometer measured levels of physical activity and sedentary time between obese and non‐obese children and adolescents: a systematic review. BMC Pediatr. 2018;18(1):106. 29523101 10.1186/s12887-018-1031-0 PMC5844092

[9] Trinh A , Campbell M , Ukoumunne OC , Gerner B , Wake M . Physical activity and 3‐year BMI change in overweight and obese children. Pediatrics. 2013;131(2):e470‐e477. 23319527 10.1542/peds.2012-1092

[10] Griffiths LJ , Sera F , Cortina‐Borja M , Law C , Ness A , Dezateux C . Objectively measured physical activity and sedentary time: cross‐sectional and prospective associations with adiposity in the Millennium Cohort Study. BMJ Open. 2016;6(4):e010366. 10.1136/bmjopen-2015-010366 PMC4838720 27067891

[11] Van Hecke L , Loyen A , Verloigne M , et al. Variation in population levels of physical activity in European children and adolescents according to cross‐European studies: a systematic literature review within DEDIPAC. Int J Behav Nutr Phys Act. 2016;13(1):70‐70. 27350134 10.1186/s12966-016-0396-4 PMC5399406

[12] U.S. Department of Health and Human Services . Physical Activity Guidelines for Americans. 2nd ed. Washington, DC: U.S. Department of Health and Human Services; 2018.

[13] Farooq MA , Parkinson KN , Adamson AJ , et al. Timing of the decline in physical activity in childhood and adolescence: Gateshead Millennium Cohort Study. Br J Sports Med. 2017;13:13. 10.1136/bjsports-2016-096933 PMC6204977 28288966

[14] Janz KF , Letuchy EM , Francis SL , Metcalf KM , Burns TL , Levy SM . Objectively measured physical activity predicts hip and spine bone mineral content in children and adolescents ages 5‐15 years: Iowa bone development study. Front Endocrinol (Lausanne). 2014;5:112‐112. 25076937 10.3389/fendo.2014.00112 PMC4097953

[15] Metcalf BS , Hosking J , Jeffery AN , Henley WE , Wilkin TJ . Exploring the adolescent fall in physical activity: A 10‐yr Cohort Study (EarlyBird 41). Med Sci Sports Exerc. 2015;47(10):2084‐2092. 25706294 10.1249/MSS.0000000000000644

[16] Cooper AR , Goodman A , Page AS , et al. Objectively measured physical activity and sedentary time in youth: the International children's accelerometry database (ICAD). Int J Behav Nutr Phys Act. 2015;12(1):113. 26377803 10.1186/s12966-015-0274-5 PMC4574095

[17] Corder K , Sharp SJ , Atkin AJ , et al. Age‐related patterns of vigorous‐intensity physical activity in youth: The International Children's Accelerometry Database. Prev Med Rep. 2016;4:17‐22. 27413656 10.1016/j.pmedr.2016.05.006 PMC4929125

[18] Dumith SC , Gigante DP , Domingues MR , Kohl HW 3rd. Physical activity change during adolescence: a systematic review and a pooled analysis. Int J Epidemiol. 2011;40(3):685‐698. 21245072 10.1093/ije/dyq272

[19] Hidding LM , Chinapaw MJM , van Poppel MNM , Mokkink LB , Altenburg TM . An updated systematic review of childhood physical activity questionnaires. Sports Med. 2018;48(12):2797‐2842. 30298479 10.1007/s40279-018-0987-0 PMC6244567

[20] Janssen I , LeBlanc AG . Systematic review of the health benefits of physical activity and fitness in school‐aged children and youth. Int J Behav Nutr Phys Act. 2010;7(1):40. 20459784 10.1186/1479-5868-7-40 PMC2885312

[21] Tanaka C , Reilly JJ , Huang WY . Longitudinal changes in objectively measured sedentary behaviour and their relationship with adiposity in children and adolescents: systematic review and evidence appraisal. Obes Rev. 2014;15(10):791‐803. 24899125 10.1111/obr.12195

[22] Higgins JPT , Green S . *Cochrane Handbook for Systematic Reviews of Interventions Version 5.1.0* The Cochrane Collaboration. http://www.handbook.cochrane.org; 2011.

[23] Drevon D , Fursa SR , Malcolm AL . Intercoder reliability and validity of WebPlotDigitizer in extracting graphed data. Behav Modif. 2017;41(2):323‐339. 27760807 10.1177/0145445516673998

[24] Tooth L , Ware R , Bain C , Purdie DM , Dobson A . Quality of reporting of observational longitudinal research. Am J Epidemiol. 2005;161(3):280‐288. 15671260 10.1093/aje/kwi042

[25] Reilly JJ , Penpraze V , Hislop J , Davies G , Grant S , Paton JY . Objective measurement of physical activity and sedentary behaviour: review with new data. Arch Dis Child. 2008;93(7):614‐619. 18305072 10.1136/adc.2007.133272

[26] Doi SAR , Barendregt JJ , Khan S , Thalib L , Williams GM . Advances in the meta‐analysis of heterogeneous clinical trials II: the quality effects model. Contemp Clin Trials. 2015;45(Pt A):123‐129. 26003432 10.1016/j.cct.2015.05.010

[27] Cuijpers P , Weitz E , Cristea IA , Twisk J . Pre‐post effect sizes should be avoided in meta‐analyses. Epidemiol Psychiatr Sci. 2016;26(4):364‐368. 27790968 10.1017/S2045796016000809 PMC6998624

[28] Cohen J . Statistical power analysis for the behavioral sciences. 2 Hillsdale, NJ: Lawrence Earlbaum Associates; 1988.

[29] Egger M , Davey Smith G , Schneider M , Minder C . Bias in meta‐analysis detected by a simple, graphical test. BMJ. 1997;315(7109):629‐634. 9310563 10.1136/bmj.315.7109.629 PMC2127453

[30] Augustin NH , Mattocks C , Cooper AR , Ness AR , Faraway JJ . Modelling fat mass as a function of weekly physical activity profiles measured by actigraph accelerometers. Physiol Meas. 2012;33(11):1831‐1839. 23110964 10.1088/0967-3334/33/11/1831

[31] Azevedo LB , Burges Watson D , Haighton C , Adams J . The effect of dance mat exergaming systems on physical activity and health‐related outcomes in secondary schools: results from a natural experiment. BMC Public Health. 2014;14(1):951. 25217144 10.1186/1471-2458-14-951 PMC4169828

[32] Butte NF , Gregorich SE , Tschann JM , et al. Longitudinal effects of parental, child and neighborhood factors on moderate‐vigorous physical activity and sedentary time in Latino children. Int J Behav Nutr Phys Act. 2014;11(1):108. 25186810 10.1186/s12966-014-0108-x PMC4174285

[33] Carver A , Timperio AF , Hesketh KD , Ridgers ND , Salmon JL , Crawford DA . How is active transport associated with children's and adolescents' physical activity over time? Int J Behav Nutr Phys Act. 2011;8(1):126. 22081977 10.1186/1479-5868-8-126 PMC3226569

[34] Cohen DA , Ghosh‐Dastidar B , Conway TL , et al. Energy balance in adolescent girls: the trial of activity for adolescent girls cohort. Obesity (Silver Spring). 2014;22(3):772‐780. 23804512 10.1002/oby.20536 PMC3825824

[35] Cohen KE , Morgan PJ , Plotnikoff RC , Callister R , Lubans DR . Physical activity and skills intervention: SCORES cluster randomized controlled trial. Med Sci Sports Exerc. 2015;47(4):765‐774. 25051389 10.1249/MSS.0000000000000452

[36] Colabianchi N , Griffin JL , McIver KL , Dowda M , Pate RR . Where are children active and does it matter for physical activity? A latent transition analysis. J Phys Act Health. 2016;13(12):1294‐1300. 27633617 10.1123/jpah.2015-0607 PMC5266643

[37] Collings PJ , Wijndaele K , Corder K , et al. Magnitude and determinants of change in objectively‐measured physical activity, sedentary time and sleep duration from ages 15 to 17.5y in UK adolescents: the ROOTS study. Int J Behav Nutr Phys Act. 2015;12(1):61. 25971606 10.1186/s12966-015-0222-4 PMC4437669

[38] Cooper AR , Jago R , Southward EF , Page AS . Active travel and physical activity across the school transition: the PEACH project. Med Sci Sports Exerc. 2012;44(10):1890‐1897. 22525779 10.1249/MSS.0b013e31825a3a1e

[39] Corder K , Sharp SJ , Atkin AJ , et al. Change in objectively measured physical activity during the transition to adolescence. Br J Sports Med. 2015;49(11):730‐736. 24273308 10.1136/bjsports-2013-093190 PMC4453714

[40] Dalene KE , Anderssen SA , Andersen LB , et al. Secular and longitudinal physical activity changes in population‐based samples of children and adolescents. Scand J Med Sci Sports. 2017;28:161. 28299832 10.1111/sms.12876

[41] Davison KK , Jago R . Change in parent and peer support across ages 9 to 15 yr and adolescent girls' physical activity. Med Sci Sports Exerc. 2009;41(9):1816‐1825. 19657287 10.1249/MSS.0b013e3181a278e2 PMC5489408

[42] De Craemer M , De Decker E , Verloigne M , De Bourdeaudhuij I , Manios Y , Cardon G . The effect of a kindergarten‐based, family‐involved intervention on objectively measured physical activity in Belgian preschool boys and girls of high and low SES: the ToyBox‐study. Int J Behav Nutr Phys Act. 2014;11(1):38. 24628972 10.1186/1479-5868-11-38 PMC3995650

[43] De Meester F , Van Dyck D , De Bourdeaudhuij I , Deforche B , Cardon G . Changes in physical activity during the transition from primary to secondary school in Belgian children: what is the role of the school environment? BMC Public Health. 2014;14(1):261. 24645802 10.1186/1471-2458-14-261 PMC3995550

[44] Dencker M , Tanha T , Wollmer P , Karlsson MK , Andersen LB , Thorsson O . Tracking of physical activity with accelerometers over a 2‐year time period. J Phys Act Health. 2013;10(2):241‐248. 22396324 10.1123/jpah.10.2.241

[45] Edwards NM , Khoury PR , Kalkwarf HJ , Woo JG , Claytor RP , Daniels SR . Tracking of accelerometer‐measured physical activity in early childhood. Pediatr Exerc Sci. 2013;25(3):487‐501. 23877325 10.1123/pes.25.3.487 PMC3782863

[46] Grydeland M , Bergh IH , Bjelland M , et al. Intervention effects on physical activity: the HEIA study—a cluster randomized controlled trial. Int J Behav Nutr Phys Act. 2013;10(1):17. 23379535 10.1186/1479-5868-10-17 PMC3598379

[47] Harding SK , Page AS , Falconer C , Cooper AR . Longitudinal changes in sedentary time and physical activity during adolescence. Int J Behav Nutr Phys Act. 2015;12(1):44. 25888805 10.1186/s12966-015-0204-6 PMC4391111

[48] Harrington DM , Davies MJ , Bodicoat DH , et al. Effectiveness of the 'Girls Active' school‐based physical activity programme: a cluster randomised controlled trial. Int J Behav Nutr Phys Act. 2018;15(1):40. 29695250 10.1186/s12966-018-0664-6 PMC5918764

[49] Jaakkola T , Hakonen H , Kankaanpaa A , et al. Longitudinal associations of fundamental movement skills with objectively measured physical activity and sedentariness during school transition from primary to lower secondary school. J Sci Med Sport. 2019;22(1):85‐90. 30098976 10.1016/j.jsams.2018.07.012

[50] Jago R , Edwards MJ , Sebire SJ , et al. Effect and cost of an after‐school dance programme on the physical activity of 11‐12 year old girls: The Bristol Girls Dance Project, a school‐based cluster randomised controlled trial. Int J Behav Nutr Phys Act. 2015;12(1):128. 26437720 10.1186/s12966-015-0289-y PMC4595057

[51] Jago R , Solomon‐Moore E , Macdonald‐Wallis C , Sebire SJ , Thompson JL , Lawlor DA . Change in children's physical activity and sedentary time between Year 1 and Year 4 of primary school in the B‐PROACT1V cohort. Int J Behav Nutr Phys Act. 2017;14(1):33. 28449679 10.1186/s12966-017-0492-0 PMC5408437

[52] Jauregui A , Villalpando S , Rangel‐Baltazar E , Lara‐Zamudio YA , Castillo‐Garcia MM . Physical activity and fat mass gain in Mexican school‐age children: a cohort study. BMC Pediatr. 2012;12:109. 22839498 10.1186/1471-2431-12-109 PMC3441390

[53] Leppanen MH , Henriksson P , Delisle Nystrom C , et al. Longitudinal physical activity, body composition, and physical fitness in preschoolers. Med Sci Sports Exerc. 2017;49(10):2078‐2085. 28538260 10.1249/MSS.0000000000001313

[54] Lima RA , Pfeiffer K , Larsen LR , et al. Physical activity and motor competence present a positive reciprocal longitudinal relationship across childhood and early adolescence. J Phys Act Health. 2017;14(6):440‐447. 28169569 10.1123/jpah.2016-0473

[55] Lipsky LM , Nansel TR , Haynie DL , et al. Diet quality of US adolescents during the transition to adulthood: Changes and predictors. Am J Clin Nutr. 2017;105(6):1424‐1432. 28446498 10.3945/ajcn.116.150029 PMC5445678

[56] Magnusson KT , Sigurgeirsson I , Sveinsson T , Johannsson E . Assessment of a two‐year school‐based physical activity intervention among 7‐9‐year‐old children. Int J Behav Nutr Phys Act. 2011;8(1):138. 22185086 10.1186/1479-5868-8-138 PMC3257198

[57] Marques A , Minderico C , Martins S , Palmeira A , Ekelund U , Sardinha LB . Cross‐sectional and prospective associations between moderate to vigorous physical activity and sedentary time with adiposity in children. Int J Obes (Lond). 2016;40(1):28‐33. 26303349 10.1038/ijo.2015.168 PMC4757733

[58] Metcalf BS , Hosking J , Jeffery AN , Voss LD , Henley W , Wilkin TJ . Fatness leads to inactivity, but inactivity does not lead to fatness: a longitudinal study in children (EarlyBird 45). Arch Dis Child. 2011;96(10):942‐947. 20573741 10.1136/adc.2009.175927

[59] Michels N , Susi K , Marques‐Vidal PM , Nydegger A , Puder JJ . Psychosocial quality‐of‐life, lifestyle and adiposity: a longitudinal study in pre‐schoolers (Ballabeina Study). Int J Behav Med. 2016;23(3):383‐392. 26809517 10.1007/s12529-016-9537-z

[60] Mitchell JA , Pate RR , Beets MW , Nader PR . Time spent in sedentary behavior and changes in childhood BMI: a longitudinal study from ages 9 to 15 years. Int J Obes (Lond). 2013;37(1):54‐60. 22430304 10.1038/ijo.2012.41

[61] Ornelas RT , Silva AM , Minderico CS , Sardinha LB . Changes in cardiorespiratory fitness predict changes in body composition from childhood to adolescence: findings from the European Youth Heart Study. Phys Sportsmed. 2011;39(2):78‐86. 21673487 10.3810/psm.2011.05.1897

[62] Owen KB , Parker PD , Astell‐Burt T , Lonsdale C . Regular physical activity and educational outcomes in youth: a longitudinal study. J Adolesc Health. 2018;62(3):334‐340. 29229462 10.1016/j.jadohealth.2017.09.014

[63] Pardo BM , Bengoechea EG , Julián Clemente JA , Lanaspa EG . Empowering adolescents to be physically active: three‐year results of the Sigue la Huella intervention. Prev Med. 2014;66:6‐11. 24811758 10.1016/j.ypmed.2014.04.023

[64] Ridgers ND , Timperio A , Crawford D , Salmon J . What factors are associated with adolescents' school break time physical activity and sedentary time? PLoS ONE. 2013;8(2):e56838. 23418606 10.1371/journal.pone.0056838 PMC3572081

[65] Santos A , Silva‐Santos S , Duncan M , Lagoa MJ , Vale S , Mota J . Relationship among changes in sedentary time, physical activity, and body mass index in young schoolchildren: A 3‐Year longitudinal study. Pediatr Exerc Sci. 2018;30(3):426‐432. 29485937 10.1123/pes.2017-0163

[66] Schwarzfischer P , Gruszfeld D , Stolarczyk A , et al. Physical activity and sedentary behavior from 6 to 11 years. Pediatrics. 2019;143(1):e20180994. 30509928 10.1542/peds.2018-0994

[67] Stefan L , Soric M , Devrnja A , Petric V , Misigoj‐Durakovic M . One‐year changes in physical activity and sedentary behavior among adolescents: the Croatian Physical Activity in Adolescence Longitudinal Study (CRO‐PALS). Int J Adolesc Med Health. 2018;0(0). 10.1515/ijamh-2017-0223 29883321

[68] Stevens J , Murray DM , Baggett CD , et al. Objectively assessed associations between physical activity and body composition in middle‐school girls: the Trial of Activity for Adolescent Girls. Am J Epidemiol. 2007;166(11):1298‐1305. 17855391 10.1093/aje/kwm202 PMC2150740

[69] Sutherland RL , Campbell EM , Lubans DR , et al. The Physical Activity 4 Everyone Cluster Randomized Trial: 2‐year outcomes of a school physical activity intervention among adolescents. Am J Prev Med. 2016;51(2):195‐205. 27103495 10.1016/j.amepre.2016.02.020

[70] Taylor RW , Murdoch L , Carter P , Gerrard DF , Williams SM , Taylor BJ . Longitudinal study of physical activity and inactivity in preschoolers: the FLAME study. Med Sci Sports Exerc. 2009;41(1):96‐102. 19092702 10.1249/MSS.0b013e3181849d81

[71] Telford RM , Telford RD , Cochrane T , Cunningham RB , Olive LS , Davey R . The influence of sport club participation on physical activity, fitness and body fat during childhood and adolescence: The LOOK Longitudinal Study. J Sci Med Sport. 2016;19(5):400‐406. 26111721 10.1016/j.jsams.2015.04.008

[72] Ten Hoor GA , Rutten GM , Breukelen GJPV , et al. Strength exercises during physical education classes in secondary schools improve body composition: a cluster randomized controlled trial. Int J Behav Nutr Phys Act. 2018;15(1):92. 30253776 10.1186/s12966-018-0727-8 PMC6156874

[73] Vaipuna TFW , Williams SM , Farmer VL , et al. Sleep patterns in children differ by ethnicity: cross‐sectional and longitudinal analyses using actigraphy. Sleep Health. 2018;4(1):81‐86. 29332685 10.1016/j.sleh.2017.10.012

[74] Väistö J , Haapala EA , Viitasalo A , et al. Longitudinal associations of physical activity and sedentary time with cardiometabolic risk factors in children. Scand J Med Sci Sports. 2019;29(1):113‐123. 30276872 10.1111/sms.13315 PMC6485341

[75] Vaitkeviciute D , Latt E , Maestu J , et al. Longitudinal associations between bone and adipose tissue biochemical markers with bone mineralization in boys during puberty. BMC Pediatr. 2016;16(1):102. 27439435 10.1186/s12887-016-0647-1 PMC4955269

[76] Van Kann DHH , Kremers SPJ , de Vries NK , de Vries SI , Jansen MWJ . The effect of a school‐centered multicomponent intervention on daily physical activity and sedentary behavior in primary school children: The Active Living study. Prev Med. 2016;89:64‐69. 27235606 10.1016/j.ypmed.2016.05.022

[77] Telford RM , Telford RD , Cunningham RB , Cochrane T , Davey R , Waddington G . Longitudinal patterns of physical activity in children aged 8 to 12 years: the LOOK study. Int J Behav Nutr Phys Act. 2013;10(1):81. 24456743 10.1186/1479-5868-10-81 PMC3691664

[78] Dewar DL , Morgan PJ , Plotnikoff RC , et al. The nutrition and enjoyable activity for teen girls study: a cluster randomized controlled trial. Am J Prev Med. 2013;45(3):313‐317. 23953358 10.1016/j.amepre.2013.04.014

[79] Ortega FB , Konstabel K , Pasquali E , et al. Objectively measured physical activity and sedentary time during childhood, adolescence and young adulthood: a cohort study. PLoS ONE. 2013;8(4):e60871. 23637772 10.1371/journal.pone.0060871 PMC3634054

[80] Brooke HL , Corder K , Griffin SJ , van Sluijs EMF . Physical activity maintenance in the transition to adolescence: a longitudinal study of the roles of sport and lifestyle activities in British youth. PLoS ONE. 2014;9(2):e89028. 24533167 10.1371/journal.pone.0089028 PMC3923069

[81] Kwon S , Janz KF , Letuchy EM , Burns TL , Levy SM . Developmental trajectories of physical activity, sports, and television viewing during childhood to young adulthood: Iowa Bone Development Study. JAMA Pediatr. 2015;169(7):666‐672. 25984811 10.1001/jamapediatrics.2015.0327 PMC4596396

[82] Kwon S , Janz KF , Letuchy EM , Burns TL , Levy SM . Active lifestyle in childhood and adolescence prevents obesity development in young adulthood. Obesity (Silver Spring). 2015;23(12):2462‐2469. 26538514 10.1002/oby.21262 PMC4701632

[83] Janssen X , Mann KD , Basterfield L , et al. Development of sedentary behavior across childhood and adolescence: longitudinal analysis of the Gateshead Millennium Study. Int J Behav Nutr Phys Act. 2016;13(1):88. 27484336 10.1186/s12966-016-0413-7 PMC4971697

[84] Dollman J , Okely AD , Hardy L , Timperio A , Salmon J , Hills AP . A hitchhiker's guide to assessing young people's physical activity: deciding what method to use. J Sci Med Sport. 2009;12(5):518‐525. 19038579 10.1016/j.jsams.2008.09.007

[85] Beltran‐Valls MR , Janssen X , Farooq A , et al. Longitudinal changes in vigorous intensity physical activity from childhood to adolescence: Gateshead Millennium Study. J Sci Med Sport. 2019;22(4):450‐455. 30448321 10.1016/j.jsams.2018.10.010

[86] WHO . Report of the commission on ending childhood obesity. 2016; 68 http://apps.who.int/iris/bitstream/10665/204176/1/9789241510066_eng.pdf?ua=1. Accessed 04 Jul, 2018.

[87] Hallal PC , Andersen LB , Bull FC , Guthold R , Haskell W , Ekelund U . Global physical activity levels: surveillance progress, pitfalls, and prospects. Lancet (London, England). 2012;380(9838):247‐257. 10.1016/S0140-6736(12)60646-1 22818937

[88] Aubert S , Barnes JD , Abdeta C , et al. Global Matrix 3.0 Physical Activity Report Card Grades for Children and Youth: Results and Analysis From 49 Countries. J Phys Act Health. 2018;15(S2):S251‐S273. 30475137 10.1123/jpah.2018-0472

