# Supplementary material for: Longitudinal changes in moderate‐to‐vigorous‐intensity physical activity in children and adolescents: A systematic review and meta‐analysis
Source: Obes Rev. 2019 Oct 23;21(1):e12953. doi: 10.1111/obr.12953 (PMC6916562; doi:10.1111/obr.12953)
Supplement: Supplementary file 1 — Table S1. Sample search strategy using OVID Table S2A. Description of included studies and methods used for objective physical activity assessment Table S2B. Moderate‐to‐vigorous physical activity (min/day) at baseline and subsequent follow up each year among boys and girls in the included studies Table S2B. Moderate‐to‐vigorous physical activity (min/day) at baseline and subsequent follow up each year among boys and girls in the included studies Table S3: Risk of Bias assessment and summary Figure S1. Contour enhanced funnel plot for random effects meta‐analysis of standardised mean MVPA difference per year in Boys and girls Table S4. Sensitivity analysis showing estimated annual MVPA change (paired standardised mean difference) according to age in boys and girls assuming correlation coefficients for 0.3, 0.5 and 0.7. Table S5. Sensitivity analysis showing estimated annual MVPA change (paired standardised mean difference) according to age in boys and girls using random effects (RE) model and quality effects (QE) model Table S6. Sensitivity analysis showing estimated annual MVPA change (paired standardised mean difference) according to age in boys and girls using random effects (RE) model and quality effects (QE) model [file OBR-21-na-s001.pdf]

Table S1. Sample search strategy using OVID

Steps	Syntax	Remarks
1	exp Cohort Studies/ or longitudinal.ti,ab. or prospective.ti,ab. or (follow?up).ti,ab.	Find all cohort studies
2	exp Clinical Trial/ or double-blind method/ or (clinical trial* or randomized controlled trial or multicenter study).pt. or exp Clinical Trials as Topic/ or ((randomi?ed adj7 trial*) or (controlled adj3 trial*) or (clinical adj2 trial*) or ((single or doubl* or tripl* or treb*) and (blind* or mask*))).ti,ab.	Find all RCTs
3	Exp Actigraphy/ or acceleromet*.ti,ab or actigraph.ti,ab or (CSA monitor).ti,ab or (activity monitor).ti,ab or (motion sensor).ti,ab	Find all studies that used accelerometer
4	exp adolescent/ or exp child/ or exp minors/ or exp students/ or adolescen$.ti,ab. or pre-adolescen.ti,ab. or youth.ti,ab. or teen.ti,ab. or (young adj child*).ti,ab. or prepubescent.ti,ab. or pubescent.ti,ab. or schoolchild*.ti,ab. or boy*.ti,ab. or girl*.ti,ab. or juvenile.ti,ab.	Find all studies of children and adolescents
5	(Moderate to vigorous physical activity).ti,ab. or MVPA.ti,ab. or (moderate-to-vigorous-physical activity).ti,ab OR (moderate adj4 vigorous)	Find moderate to vigorous physical activity
6	(1 OR 2) AND (3 AND 4 AND 5)	

Table S2A. Description of included studies and methods used for objective physical activity assessment

No	Study, year	Data source	Year of recruitment at baseline	City, Country	Cohort Name	Study Design	Brand of Accelerometer	Number of measurement days	Minimum days to consider valid	Weartime for valid day (h)	MVPA cutoff	Year PA assessment commenced	Country	Includes Weekend	Epoch Length (s)
1	Augustine et al., 2012	Email	2003	Avon, United Kingdom	ALSPAC (Avon Longitudinal Study of Parents and Children)	Cohort	ActiGraph AM 7164 and GT1M (at age 14 for some)	7	3d	10h	3200 cpm	2003	UK	Yes	60
2	Azevedo et al., 2014	Email	Sep to Dec 2010	North East of England.	Dance mats	Non RCT	ActiGraph 7164	7	3d	10h	2296 cpm	2010	UK	Yes	30
3	Butte et al., 2014	Email	2007-2009	San Francisco, USA		Cohort	Actical accelerometer	3	3d	16.6h	AEE>=0.10 for PAR>3.0	2007	USA	Yes	60
4	Carver et al., 2011	Paper	2001	Melbourne, Australia	CLAN (Children Living in Active Neighbourhoods)	Cohort	ActiGraph 7164	8	4d	10h	4 METS	2001	Australia	Yes	60
5	Cohen et al., 2014	Paper	2008	San Diego, CA suburban Minneapolis	TAAG (Trial of Activity for Adolescent Girls)	RCT	ActiGraph 7164	6	N/M	8.3h on WE and 10.3h or WD	1500 cpm	2008	USA	Yes	30
6	Cohen et al., 2015	Email	Mar 2012	Australia	SCORES (Supporting Children's Outcomes using Rewards, Exercise and Skills intervention)	RCT	Actigraph GT3X+	7	3d	8h	2296 cpm	2012	Australia	Yes	10
7	Colabianchi et al., ,2016	Paper	2010-2011	South Carolina, USA	TRACK (Transitions and Activity Changes in Kids)	Cohort	ActiGraph GT1M and GT3X	7	2d	8h	Freedson	2010	USA	Yes	60
8	Collings et al., 2015	Email	Apr-Jul 2005	Cambridge-Shire	ROOTS	Cohort	Actiheart	4	N/M	N/M	4 METS	2005	UK	Yes	30
9	Cooper et al., 2012	Paper	September 2006	Bristol, UK	PEACH (Personal and Environmental Associations with Children's Health)	Cohort	GT1M; ActiGraph LLC, Pensacola	7	N/M	N/M	2296 cpm	2006	UK	Yes	N/M
10	Corder et al., 2015	Email	2007	Norfolk East London	SPEEDY (Sport, Physical activity and Eating behaviour: Environmental Determinants in Young People)	Cohort	ActiGraph accelerometers (GT1M)	7	3d	8.3h	2000 cpm	2007	UK	Yes	5
11	Dalene et al., 2017	Paper	2005	Norway	PANCS (Physical Activity among Norwegian Children Study)	Cohort	Actigraph 7164,GT1,GT3X+	4d at baseline 7 d post	2d	8h	2000 cpm	2005	USA	Yes	10
12	Davison et al., 2009	Paper	N/M	Pennsylvania		Cohort	ActiGraph 7164	7	4d	10h	Freedson		USA	Yes	30
13	De Craemer et al., 2014	Email	March to Jun 2012	North Belgium	The Toy Box study	RCT	ActiGraph GT1M, GT3X and GT3X +	6	3d	6h	2296 cpm	2012	Belgium	Yes	15
14	De Meester et al., 2014	Paper	2009-2010	East-West Flanders, Belgium		Cohort	ActiGraph GT1	7	3d	10h	3200 cpm	2009	Belgium	No	60
15	Dencker et al., 2013	Email	Not mentioned	Malmo, Sweden		Cohort	MTI models 7164	4	3d	8h	3500 cpm		Sweden	Yes	10
16	Dewar et al., 2014	Email	May 2010	Newcastle, Australia	NEAT (Nutrition and Enjoyable Activity for Teen Girls Study)	RCT	MTI models 7164; GT1M, GT3X	7	3d	10h	Freedson	2010	Australia	Yes	30
17	Edwards et al., 2013	Paper	2001	Cincinnati, USA	Cincinnati Children's Hospital Medical Center (CCHMC)	Cohort	RT3	3	1d	8h	1400 cpm	2001	USA	Yes	60
18	Farooq et al., 2017	Email	Oct 2006 to Dec 2007	North East of England.	GMS (The Gateshead Millennium Study)	Cohort	Actigraph GT1M	7	3d	6h	2296 cpm	2006	UK	Yes	15
19	Grydeland et al., 2013	Paper	Sep 2007	Norway	HEIA (HEalth in Adolescents)	RCT	ActiGraph 7164 and GT1M	5	3d	8h	2000cpm	2007	Norway	Yes	10
20	Harding et al., 2015	Email	2008/2009	Bristol, UK	PEACH (Personal and Environmental Associations with Children's Health)	Cohort	ActiGraph GT1M	7	N/M	N/M	2296 cpm	2008	UK	Yes	N/M
21	Harrington et al., 2018	Email	Spring 2015	Leicester City, Leicestershire and Rutland (LLR), UK	Girls Active	RCT	GENEActiv	7	2d	>16h	N/M or referenced	2015	UK	Yes	N/M
22	Jaakkola et al., 2019	Email	2013-2014	Finland	Finnish Schools on the Move	Cohort	Actigraph GT3X+ or wGT3X+	7	3d	8.3h	2296 cpm	2013	Finland	Yes	15
23	Jago et al., 2015	Paper	Nov 2013	Bristol, UK	Bristol Girls Dance Project	RCT	ActiGraph GT3X+	7	3d	8.3h	2296 cpm	2013	UK	Yes	N/M
24	Jago et al., 2017	Paper	Feb 2012-Jul 2013	Bristol, UK	B-PROACTIV	Cohort	ActiGraph wGTX3	5	2 d(1weekend)	8.3h	2296 cpm	2012	UK	Yes	N/M
25	Janz et al., 2014	Paper	1998	Iowa	IBDS (Iowa Bone Development Study)	Cohort	ActiGraph GT1M, GT3	4-5d	3d	10h	2296 cpm	1998	USA	Yes	60
26	Jauregui et al., 2012	Email	Mar 2004	Cuernavaca, Mexico.		Cohort	RT3	5	1d (avg 4d)	avg 13.6h/d	970 cpm	2004	Mexico	Yes	60
27	Leppanen et al., 2017	Email	2014-2016	Sweden	MINISTOP	RCT	Actigraph wGT3x-BT	7	3d	10h	818‡	2014	Sweden	Yes	10
28	Lima et al., 2017	Email	2001	Copenhagen	Copenhagen School Child Intervention Study (CoSCIS)	Cohort	ActiGraph 7164, GTIM	4	3d	8h	1500 cpm	2001	Denmark	Yes	10
29	Lipsky et al., 2017	Email	2010	Nationally representative sample of USA	The NEXT Plus	Cohort	ActiGraph GT3X	7	4d	8.3h	2296 cpm	2010	USA	Yes	30
30	Magnusson et al., 2011	Paper	2006	Reykjavik, Iceland		RCT	ActiGraph GT1M	7	2d	10h	2000 cpm	2006	Iceland	No	60
31	Marques et al., 2016	Paper	2010	Oeiras Municipality, in Lisbon Metropolitan area, Portugal.		Cohort	ActiGraph GT1M	N/M	3d	10h	4 METS	2010	Portugal	Yes	15
32	Metcalf et al., 2011	Email	Jan 2000 to Jan 2001	Plymouth, UK	The EarlyBird study	Cohort	Actigraph accelerometers (formerly MTI/CSA)	7	5 d (1 weekend day)	9h	N/M or referenced	2000	UK	Yes	10
33	Michels et al., 2016	Email	2008	Switzerland	The Ballabeina study	Cohort	ActiGraph MT1/CSA 7164;	5	3d	6h	2292 cpm	2008	Switzerland	Yes	15
34	Mitchel et al., 2013	Paper	2000	(1) Little Rock, Arkansas; (2) Irvine, California; (3) Lawrence, Kansas; (4) Boston, Massachusetts; (5) Philadelphia, Pennsylvania; (6) Pittsburgh, Pennsylvania; (7) Charlottesville, Virginia; (8) Seattle, Washington; (9) Hickory and Morganton, North Carolina; and (10) Madison, Wisconsin, USA	National Institute of Child Health and Human Development (NICHD) Study of Early Child Care and Youth Development	Cohort	ActiGraph 7164, ActiGraph GTM1	7	3d	10h	2296 cpm	2000	USA	Yes	60
35	Ornelas et al., 2011	Paper	2000	Madeira, Portugal		Cohort	WAM 7164, CSA, GT1M ActiGraph	3	3d	N/M	2000 cpm	2000	Portugal	Yes	60
36	Ortega et al., 2013	Paper	1998	Tartu city (Estonia)	EYHS (The European Youth Heart Study)	Cohort	ActiGraph AM7164 at baseline and GT1M at followup	4	3d	10h	2000 cpm	1998	Estonia	Yes	60
37	Owen et al., 2018	Email	Jan-Apr 2014	Western Sydney	AMPED (Activity and Motivation in Phyiscal Education)	Cohort	Actigraph wGT3x+	7	1d	8h	2296 cpm	2014	Australia	Yes	1
38	Pardo et al., 2014	Paper	Oct 2009	Huesca, Spain	Sigue la Huella	Quasi-experimental	ActiGraph GT1M	7	4d	10h	2292 cpm	2009	Spain	Yes	30
39	Ridgers et al., 2013	Email	2006	Melbourne, Australia	HEAPS (Healthy Eating and Play Study)	Cohort	ActiGraph 7164	8	3d	10h	4 METS	2006	Australia	Yes	60
40	Santos et al., 2018	Paper	2009-2010	Porto, Portugal		Cohort	Actigraph, GT1M	7	4d	10h	2296 cpm	2009	Portugal	Yes	5
41	Schwarzfischer et al., 2019	Email	2008-2014	5 European countries: Germany, Italy, Belgium, Poland, and Spain.	Childhood Obesity Project	Longitudinal study (	SenseWear Armband 2	3	2d	N/M	4 METS	2008	European countries	No	N/M
42	Stefan et al., 2018	Paper	Mar & Jun 2014	Zagreb (Croatia)	CROPALS (Croatian Physical Activity inAdolescence Longitudina	Cohort	SenseWear Armband	5	3d	10h	4 METS	2014	Croatia	Yes	N/M
43	Stevens et al., 2007	Paper	Spring 2003	Tucson, Arizona; San Diego, California; New Orleans, Louisiana; Baltimore, Maryland/Washington, DC; Minneapolis, Minnesota; and Columbia, South Carolina.	TAAG (Trial of Activity for Adolescent Girls)	RCT	using Actigraph accelerometers (MTI model 7164	6	6d	at least 80% of 18h	4.6 METS	2003	USA	Yes	30
44	Sutherland et al., 2016	Email	Mar to Jun 2012	NSW, Disadvantaged schools, Australia	PA4E1 (Physical Activity for Every One)	RCT	ActiGraph GT3X+ and GT3X	7	3d	10h	2296 cpm	2012	Australia	Yes	15
45	Taylor et al., 2009	Paper	N/M	Dunedin, New Zealand	FLAMES (Family, Lifestyle, Activity, Movement, and Eating Study)	Cohort	Mini-Mitter (Bend, OR) omnidirectional Actical accelerometers	5	N/M	N/M	0.04 kcal/kg/min		New Zealand	No	N/M
46	Telford et al., 2013	Paper	Sep to Dec 2005	Canberra, Australia	LOOK (Lifestyle of Our Kids)	Cohort	ActiGraph GT1M	7	N/M	10h	2296 cpm	2005	Australia	Yes	60
47	Telford et al., 2015	Paper	Sep to Dec 2005	Canberra, Australia	LOOK (Lifestyle of Our Kids)	Cohort	ActiGraph GT1M	7	N/M	10h	2296 cpm	2005	Australia	Yes	60
48	Ten Hoor et al., 2018	Email	Mar 2015	Dutch, Netherlands		RCT	Actigraph GT3x	5	3d	8h	2296	2015	Netherlands	Yes	N/M
49	Vaipuna et al., 2018	Email	March 2010 to Dec 2011 & March 2012 to Dec 2013	New Zealand	PLAY	RCT	Actigraph GT3x	8	2d	8h	2296	2010	New Zealand	Yes	15
50	Vaisto et al., 2019	Email	2007-2009	Kuopio, Finland	Physical activity and nutrition in children (PANIC)	Cohort	Actiheart	4	>~4.1d	12h	4 METS	2007	Finland	Yes	60
51	Vaitkeviciute et al., 2014	Paper	2010	Tartu city (Estonia)		Cohort	GT1M (ActiGraph, Pensacola, USA)	7	3d	8h	2000 cpm	2010	Estonia	Yes	15
52	Vann Kann et al., 2016	Email	Mar-Jun 2013	Southern Limburg Netherlands	The Active Living	Quasi-experimental	Actigraph GT3X+	5	1d	8h	2296 cpm	2013	Netherlands	No	10

N/M: Not mentioned; ‡ Wrist worn

Table S2B. Moderate-to-vigorous physical activity (min/day) at baseline and subsequent follow up each year among boys and girls in the included studies

			Baseline					After 1 yr		After 2 yr		After 3 yr		After 4 yr		After 5 yr		After 6 yr		After 7 yr		After 8 yr		After 9 yr		After 10 yr		After 12 year	
No	Study, year	Gender	N	age	Region	Age (mean±SD) [Median]	MVPA min/day	N	MVPA min/day	N	MVPA min/day	N	MVPA min/day	N	MVPA min/day	N	MVPA min/day	N	MVPA min/day	N	MVPA min/day	N	MVPA min/day	N	MVPA min/day	N	MVPA min/day	N	MVPA min/day
1	Augustine et al., 2012	Boys	1715	12	2003	11.75±0.22	28.41±17.25			2022	28.88±18.81			1048	29.79±20.45														
		Girls	3117	12		11.79±0.25	18.20±11.86			2297	19.67±14.62			1309	18.37±15.34														
2	Azevedo et al., 2014	Boys	26	11	2010	11.3±0.4	70.91±20.67	26	65.75±23.81																				
		Girls	43	11		11.3±0.4	56.25±19.47	43	49.98±20.60																				
3	Butte et al., 2014	Boys	40	8	2007	8	72.0±35.1	40	73.2±32.9	40	67.7±30.9																		
		Girls	46	8		8	66.2±60.8	46	48.0±22.8	46	39.1±21.8																		
		Boys	33	9		9	84.9±53.6	33	62.7±31.4	33	68.3±34.7																		
		Girls	38	9		9	46.9±27.2	38	42.0±23.7	38	44.2±24.0																		
		Boys	19	10		10	60.7±30.4	19	57.6±23.6	19	56.7±34.1																		
		Girls	27	10		10	47.2±23.8	27	39.1±22.3	27	42.5±27.8																		
4	Carver et al., 2011	Boys	71	6	2001		154.2±34.4					71	117.5±31.6			71	85.2±29.4												
		Girls	63	6			131.8±30.4					63	88.6±23.7			63	57.0±20.4												
5	Cohen et al., 2014	Girls	265	14	2008	13.9±0.4	Mod: 17.2±6.7<br>Vig: 5.0±4.5			265	Mod: 17.4±8.5<br>Vig: 3.6±4.2	265	Mod: 16.3±8.2<br>Vig: 3.3±4.6																
6	Cohen et al., 2015	Boys	12	8	2012	8	67.5±17.5	12	54.6±16.8																				
		Boys	14	9		9	68.1±17.5	14	58.6±14.5																				
		Girls	16	8		8	48.9±16.9	16	37.2±12.0																				
		Girls	22	9		9	46.7±17.0	22	40.0±16.1																				
7	Colabianchi et al., 2016	Boys	233	10	USA	10.6	46.1±27.1 SE	233	39.1±22.7 SE																				
		Girls	287	10		10.6	26.9±13.9 SE	287	21.4±12.4 SE																				
8	Collings et al., 2015	Boys	72	15	2005	15.1±0.3	81.7±38.5					72	71.3±36.1																
		Girls	72	15		15.1±0.3	43.7±30.0					72	36.5±25.2																
9	Cooper et al., 2012	Boys	250	11	2006	11.0±0.4	70.1+24.1†	257	69.8+24.0†																				
		Girls	315	11		11.0±0.4	56.0+17.6†	313	58.2+21.8†																				
10	Corder et al., 2015	Boys	377	10	2007	10.2±0.3	84.1±24.5	377	81.8±23.1					153	66.8±25.7														
		Girls	528	10		10.3±0.3	66.8±20.2	528	64.4±19.8					187	58.5±23.0														
11	Dalene et al., 2017	Girls	272	9	2005	9.6±0.4	78±2.3 SE											272	60±2.3 SE										
		Boys	286	9		9.7±0.4	96±2.7 SE											286	70±1.9 SE										
12	Davison et al., 2009	Girls	96	13	0	13	35.575±12.92			96	26.25±11.14																		
13	De Craemer et al., 2014	Boys	92	4	2012	4.43±0.55	57.33±25.39	92	58.6±26.78																				
		Girls	79	4		4.43±0.55	55.47±20.48	79	56.49±19.04																				
14	De Meester et al., 2014	Boys	65	11	2009	11.1±0.5	34.55±20.60			65	40.79±26.57																		
		Girls	58	11		11.1±0.5	19.78±13.65			58	21.59±15.40																		
15	Dencker et al., 2013	Boys	46	9	0	9	45.9±20.3	46	45.2±19.2																				
		Girls	49	9		9	31.8±13.0	49	33.5±13.3																				
		Boys	44	10		10	43.3±20.7	44	41.3±19.5																				
		Girls	28	10		10	38.0±14.6	28	41.7±24.8																				
16	Dewar et al., 2014	Girls	139	13	2010	13	36.4±15.4	139	33.5±17.4	97	33.2±18.6																		
17	Edwards et al., 2013	Boys	124	3	2001	3.4±0.3	93		99		103		94		89														
		Girls	110	3		3.4±0.3	78		80		82		79		68														
18	Farooq et al., 2017	Boys	217	7	2006	7.5±0.5	75.5±25.5			209	70.0±23.9					176	60.3±24.4					131	51.4±22.4						
		Girls	214	7		7.5±0.4	63.4±20.9			219	56.4±19.8					209	47.4±20.9					147	41.0±16.8						
19	Grydeland et al., 2013	Boys	308	11	2007	11.2±0.3	75±SE1.7	308	81±SE2.2																				
		Girls	392	11		11.2±0.3	62±SE1.2	392	62±SE1.4																				
20	Harding et al., 2015	Boys	140	12	2008	12.0±0.4	60.4±26.27					140	57.46±28.0																
		Girls	223	12		12.0±0.4	46.46±18.68					223	45.49±25.87																
21	Harrington et al., 2018	Girls	134	11	2015	11	38.4±8.0	134	36.2±8.0																				
		Girls	225	12		12	37.8±9.7	225	36.0±9.4																				
		Girls	153	13		13	34.4±7.4	153	32.2±7.1																				
		Girls	50	14		14	33.8±6.8	50	30.3±7.3																				
22	Jaakkola et al., 2019	Boys	39	12	2013	12.03±0.38	62.88±24.82	39	60.42±28.65																				
		Girls	60	12		12.03±0.38	48.18±18.95	60	43.90±19.08																				
23	Jago et al., 2015	Girls	280	11	2013	11-12y	49.15 [37.38-60.64]†<br>32.5 [22.17-26.16]‡	262	53.15±19.61†<br>36.56±26.16‡																				
24	Jago et al., 2017	Boys	899	5	2012	5-6	72.4±20.7					899	69.3±22.7																
		Girls	938	5		5-6	70.9±21.1					938	55.6±17.3																
25	Janz et al., 2014	Boys	184	5	1998	5.2±0.4	59.5±23.7					245	65.4±28.7					250	64.1±28.6			243	52.1±25.3			212	38.0±20.3	192	35.8±21.4
		Girls	203	5		5.3±0.4	47.7±19.8					248	46.3±20.3					247	39.7±19.2			238	32.9±19.3			204	25.7±16.7	195	23.7±15.5
26	Jauregui et al., 2012	Boys	87	6	2004	5.9±0.4	163±58			87	143±53																		
		Girls	118	6		6.0±0.4	125±45			118	98±46																		
27	Leppanen et al., 2017	Boys	73	4	2014	4.5±0.15	101.4±27.9	73	116.4±28.3																				
		Girls	62	4		4.5±0.16	101.6±21.4	62	116.3±23.1																				
28	Lima et al., 2017	Boys	31	6	2001	6.8±0.3	85.8±34.2					32	72.1±21.2									32	48.6±24.6						
		Girls	30	6		6.6±0.4	71.9±17.3					32	63.97±20.9									32	57.3±22.14						
29	Lipsky et al., 2017	Boys	257	16	2010	16.5	30.4± 1.3SE	257	33.3± 1.5 SE	257	34.4±1.4 SE	257	33.2±1.8 SE																
		Girls	309	16		16.5	20.7± 0.9SE	309	21.0±0.9 SE	309	20.8±1.0 SE	309	19.7±1.1 SE																
30	Magnusson et al., 2011	Boys	39	7	2006	[7.3]	[40.7]<br>{40.8}		[39.0]<br>{50}		[44.0]<br>{44.0}																		
		Girls	57	7		[7.4]	[26.8]<br>{29.1}		[24]<br>{21}		[28]<br>{26.5}																		
31	Marques et al., 2016	Boys	258	10	2010	10.2±0.9	67.8±25.6			235	64.2±25.3																		
		Girls	252	10		10.0±0.7	51.4±18.1			262	44.8±20.6																		
32	Metcalf et al., 2011	Boys	153	5	2000	5	54	135	57	132	59	129	58	138	61	133	57	131	55	122	56	124	56	129	53	108	50		
		Girls	120	5		5	44	113	49	97	46	96	48	135	43	132	39	125	35	122	35	126	36	114	35	98	32		
33	Michels et al., 2016	Boys	81	4	2008	4	36.77±8.35	81	41.97±10.05																				
		Girls	68	4		4	33.85±10.18	68	35.53±9.15																				
		Boys	97	5		5	36.51±8.89	97	43.73±10.27																				
		Girls	121	5		5	33.74±8.73	97	38.85±9.68																				
34	Mitchel et al., 2013	Boys	391	9	2000	9	59.7[41.4-79.6]			369	46.7[32.0-62.3]	286	38.4[26.3-54.7]					257	27.6[17.1-42.6]										
		Girls	407	9		9	43.7[32.9-57.6]			382	32.4[23.7-43.9]	290	27.2[18.6-37.9]					217	14.9[9.4-24.9]										
35	Ornelas et al., 2011	Boys	42	10	2000	9.8±0.3	193.1±73.2															189.1±109.7							
		Girls	44	10		9.8±0.3	154.9±47.2															110.1±67.9							
36	Ortega et al., 2013	Boys	214	9	1998	9.6±0.5	79.8±55.1																	114	59.1±30.7				
		Boys	180	9		9.6±0.3	99.9±48.0											107	52.4±23.1										
		Girls	220	9		9.4±0.5	60.8±42.8																	149	52.3±29.6				
		Girls	213	9		9.5±0.4	73.3±30.4											133	44.2±19.7										
37	Owen et al., 2018	Boys	133	13	2014	12.98±0.53	55.55±19.42	133	52.86±23.66																				
		Girls	107	13		12.86±0.54	42.64±14.96	107	39.99±16.49																				
38	Pardo et al., 2014	Girls	53	12	2009	12	40.0±16.5	51	49.5±17.9	41	44.0±15.6																		
		Boys	47	12		12	58.7±21.1	37	69.3±25.0	35	40.0±16.9																		
		Girls	37	12		12	51.9±22.0	28	45.5±19.5																				
		Boys	43	12		12	69.8±32.9	24	70.0±30.8																				
39	Ridgers et al., 2013	Boys	30	13	2006	13	52.9±22.8			30	45.9±20.6																		
		Girls	26	13		13	33.7±17.5			26	27.9±13.5																		
		Boys	26	14		14	36.2±17.9			26	37.0±21.0																		

Table S2B. Moderate-to-vigorous physical activity (min/day) at baseline and subsequent follow up each year among boys and girls in the included studies

			Baseline					After 1 yr		After 2 yr		After 3 yr		After 4 yr		After 5 yr		After 6 yr		After 7 yr		After 8 yr		After 9 yr		After 10 yr		After 12 year	
No	Study, year	Gender	N	age	Region	Age (mean±SD) [Median]	MVPA min/day	N	MVPA min/day	N	MVPA min/day	N	MVPA min/day	N	MVPA min/day	N	MVPA min/day	N	MVPA min/day	N	MVPA min/day	N	MVPA min/day	N	MVPA min/day	N	MVPA min/day	N	MVPA min/day
		Girls	29	14		14	23.7±13.9			29	20.3±14.5																		
40	Santos et al., 2018	Boys	28	6	2009	6.3±0.3	74.7±13.8					28	76.2±19.1																
		Girls	36	6		6.3±0.3	60.8±14.1					36	56.7±18.9																
41	Schwarzfischer et al., 2019	Boys	136	6	2008	6	126.02±59.79			136	146.70±71.65					141	108.77±53.77												
		Girls	161	6		6	102.25±58.93			161	98.65±64.41					162	67.14±48.38												
42	Stefan et al., 2018	Boys	28	15	2014	15.5±0.3	Mod:121±51 Vig:22	28	Mod:72±29 Vig:8																				
		Girls	53	15		15.6±0.4	Mod:79±29 Vig:5	53	Mod:58±41 Vig:2																				
43	Stevens et al., 2007	Girls	984	12	2003	11.9±0.4	23.5±11.6			984	22.0±10.8																		
44	Sutherland et al., 2016	Boys	239	12	2012	12	61.03±22.82	124	62.24±23.8	82	66.48±39.16																		
		Girls	254	12		12	46.97±16.30	160	45.75±16.29	109	45.06±18.52																		
45	Taylor et al., 2009	Girls	95	3	0	3	44±53	79	17±16	81	23±24																		
		Boys	113	3		3	40±55	101	16±20	105	21±23																		
46 & 47	Telford et al., 2013 & 2015	Boys	282	11	Australia	11.1±0.4	43(5th 6, 95th 116)	270	43(5th 6, 95th 110)							81	26.34												
		Girls	266	11		11.1±0.4	31(5th 5, 95th 88)	265	30(5th 4, 95th 81)							76	20.69												
48	Ten Hoor et al., 2018	Boys	24	12	2015	12	39.86±23.65	24	30.84±18.56																				
		Girls	41	12		12	25.77±16.89	41	25.5±16.14																				
		Boys	15	13		13	31.13±19.71	15	29.22±19.01																				
		Girls	18	13		13	25.6±10.94	18	19.56±12.21																				
49	Vaipuna et al., 2018	Boys	53	6	2010	6.54±0.26	64.5±24.4	53	73.4±32.9	41	63.5±26.1																		
		Boys	33	7		7.4±0.3	60.6±22.8	33	77.24±28.30	29	57.53±35.27																		
		Boys	58	8		8.5±0.23	61.25±31.51	58	72.93±31.3	46	69.82±33.45																		
		Boys	23	9		9.4±0.29	64.77±32.2	23	57.74±21.23	17	50.21±29.6																		
		Girls	55	6		6.54±0.27	57.54±24.67	55	57.42±24.87	48	57.96±24.32																		
		Girls	49	7		7.43±0.27	54.21±22.30	49	66.28±30.84	46	53.91±24.02																		
		Girls	66	8		8.52±0.28	46.68±19.85	66	57.95±32.85	56	45.29±22.53																		
		Girls	28	9		9.36±0.29	60.86±29.75	28	48.78±24.12	21	48.77±27.72																		
50	Vaisto et al., 2019	Boys	147	7	2007	7.7±0.4	129.4±67.5			147	121.3±59.5																		
		Girls	165	7		7.6±0.4	96.0±54.3			165	77.4±40.5																		
51	Vaitkeviciute et al., 2014	Boys	96	12	2010	12.1±0.95	64.5 ± 23.5	96	60.4 ± 25.8	96	56.5 ± 26.1																		
52	Vann Kann et al., 2016	Boys	42	9	2013	9	66.54±20.59	21	62.77±19.26																				
		Girls	76	9		9	53.65±23.15	52	46.47±19.07																				
		Boys	52	10		10	63.58±24.05	27	64.62±30.46																				
		Girls	72	10		10	52.98±19.40	36	44.71±19.00																				

† Weekdays; ‡ Weekends; [at school]; {after school}; Median [IQR]; mean±SD

Table S3: Risk of Bias assessment and summary

Included studies	Selection Bias	Performance Bias (Accelerometer)	Detection Bias (MVPA)	Detection bias (MVPA change/year)	Attrition Bias (MVPA)	Attrition Bias (MVPA Change/year)	Selective Reporting Bias	Other Sources of Bias
Augustin et al., 2012	+	+	+	+	+	+	+	+
Azevedo et al.	?	+	+	+	–		+	+
Butte et al., 2014	?	?	+	+	+	+	+	+
Carver et al., 2011	?	+	+	+	–		+	+
Cohen et al., 2014	+	?	+	–	+		+	+
Cohen et al., 2015	?	+	+	+	–	–	+	+
Colabianchiet et al., 2016	?	?	+	–	+	+	+	+
Collings et al., 2015	+	–	+	+	–		+	+
Cooper et al., 2012	?	+	–	–	–	–	+	+
Corder et al., 2015	+	–	+	+	–	–	+	+
Dalene et al., 2017	+	?	+	+	–	–	+	+
Davison et al., 2009	+	+	+	–	+		+	+
De Craemer et al., 2014	+	?	+	+	–	–	+	+
De Meester et al., 2014	?	+	+		–		+	+
Dencker et al., 2013	?	+	+		?		+	+
Dewar et al., 2013	+	+	+	+	+	+	+	+
Edwards et al., 2013	–	–	+	–	–	–	+	+
Farooq et al., 2017	+	?	+		+		+	+
Grydeland et al., 2013	?	+	+		+		+	+
Harding et al., 2015	+	–	+	+	–		+	+
Harrington et al., 2018	+	?	+	+	–	–	+	+
Jaakkola et al., 2019	+	+	+	+	–	–	+	+
Jago et al., 2015	+	+	+	–	+	+	+	+
Jago et al., 2017	?	?	+		–		+	+
Janz et al., 2014	+	+	+		–		+	+
Jauregui et al., 2012	–	?	+	+	?	?	+	+
Leppanen et al., 2017	?	+	+	+	+	+	+	+
Lima et al., 2017	?	+	+		–		+	+
Lipsky et al., 2017	+	+	+	+	?		+	+
Magnusson et al., 2011	–	?	–	–	–	–	+	+
Marquest et al., 2016	+	–	+		+		+	+
Metcalf et al., 2011	+	+	+	+	–	–	+	+
Michels et al., 2016	+	+	+	+	?	?	+	+
Mitchell et al., 2013	+	+	+	–	–	–	+	+
Orlenas et al., 2011	+	–	+		–		+	+
Ortega et al., 2013	+	+	+		–		+	+
Owen et al., 2018	–	–	+	+	+	+	+	+
Pardo et al., 2014	–	+	+		+	+	+	+
Ridgers et al., 2013	+	+	+		–		+	+
Santos et al., 2018	–	+	+		+		+	+
Schwarzfischer., 2019	+	–	+		?		+	+
Stefan et al., 2018	+	+	+	+	–	–	+	+
Stevens et al., 2007	+	+	+		+		+	+
Sutherland et al., 2016	–	+	+	+	–	–	+	+
Taylor et al., 2009	+	–	+	+	+	+	+	+
Telford et al., 2015	+	?	+	–	+	+	+	+
Ten Hoor et al., 2018	?	+	+	+	–	–	+	+
Vaipuna et al., 2018	–	?	+	+	–	–	+	+
Vaisto et al. 2019	+	+	+		–		+	+
Vaitkeviciute et al., 2016	–	+	+	+	–	–	+	+
Van Kann et al., 2016	–	–	+	+	?	?	+	+

Summary	Selection Bias	Performance Bias (Accelerometer)	Detection Bias (MVPA)	Detection bias (MVPA change/year)	Attrition Bias (MVPA)	Attrition Bias (MVPA Change/year)	Selective Reporting Bias	Other Sources of Bias
– High	20%	20%	4%	26%	55%	55%	0%	0%
? Unclear	25%	24%	0%	0%	12%	10%	0%	0%
+ Low	55%	57%	96%	74%	33%	34%	100%	100%

Figure S1. Contour enhanced funnel plot for random effects meta-analysis of standardised mean MVPA differnce per year in Boys and girls

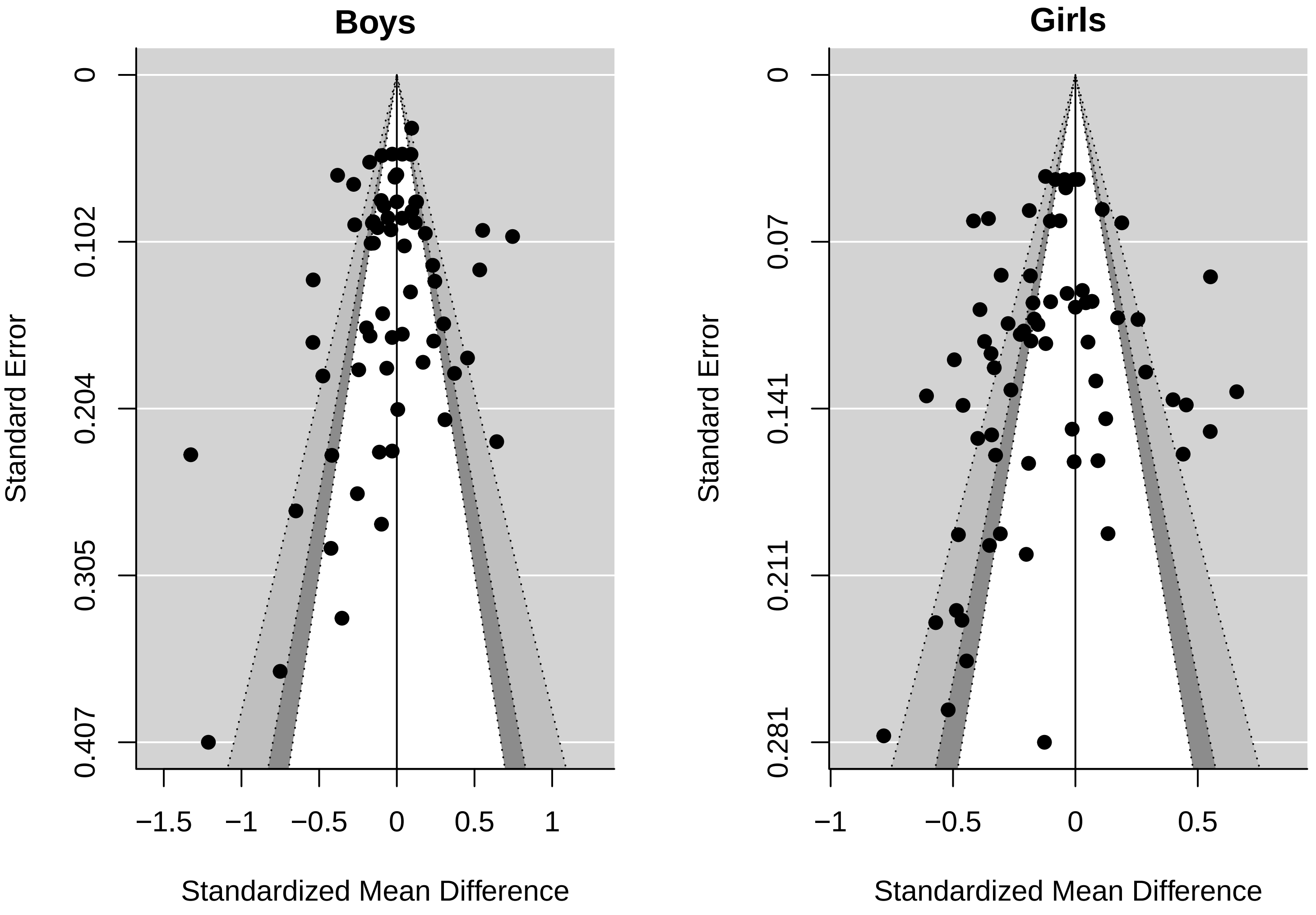

Table S4. Sensitivity analysis showing estimated annual MVPA change (paired standardised mean difference) according to age in boys and girls assuming correlation coefficients for 0.3, 0.5 and 0.7.

	Boys						Girls					
	r=0.3		r=0.5		r=0.7		r=0.3		r=0.5		r=0.7	
Age (years)	SMD (95% CI)	I^2	SMD (95% CI)	I^2	SMD (95% CI)	I^2	SMD (95% CI)	I^2	SMD (95% CI)	I^2	SMD (95% CI)	I^2
**3**	-0.14(-0.8 to 0.5)	95.2	-0.14(-0.8 to 0.5)	95.2	-0.14(-0.8 to 0.5)	95.2	-0.24(-0.8 to 0.4)	93.6	-0.24(-0.8 to 0.4)	93.8	-0.23(-0.9 to 0.4)	94.2
**4**	**0.31(0.1 to 0.5)**	84.6	**0.31(0.1 to 0.5)**	84.0	**0.32(0.1 to 0.6)**	82.8	**0.24(0.0 to 0.4)**	75.8	**0.24(0.0 to 0.4)**	75.0	**0.25(0.1 to 0.4)**	73.5
**5**	0.25(-0.3 to 0.8)	96.3	0.24(-0.3 to 0.8)	96.5	0.21(-0.4 to 0.8)	96.9	0.26(-0.1 to 0.7)	92.6	0.25(-0.2 to 0.7)	92.9	0.24(-0.2 to 0.7)	93.5
**6**	0.10(-0.1 to 0.3)	65.6	0.10(-0.1 to 0.3)	69.6	0.09(-0.1 to 0.3)	75.5	**-0.19(-0.3 to 0.0)**	46.4	**-0.21(-0.4 to 0.0)**	60.3	-0.24(-0.5 to 0.0)	75.6
**7**	0.09(-0.2 to 0.4)	77.8	0.10(-0.2 to 0.4)	78.6	0.11(-0.2 to 0.4)	80.1	-0.01(-0.3 to 0.3)	82.5	-0.03(-0.4 to 0.3)	84.9	-0.06(-0.4 to 0.3)	88.2
**8**	-0.09(-0.4 to 0.2)	77.4	-0.09(-0.4 to 0.2)	77.4	-0.09(-0.4 to 0.2)	77.4	-0.11(-0.4 to 0.2)	86.5	-0.11(-0.4 to 0.2)	87.6	-0.10(-0.5 to 0.3)	89.2
**9**	**-0.19(-0.3 to -0.1)**	0.0	**-0.20(-0.3 to -0.1)**	0.3	**-0.23(-0.4 to -0.1)**	21.3	**-0.28(-0.4 to -0.2)**	10.1	**-0.29(-0.4 to -0.2)**	13.9	**-0.32(-0.5 to -0.2)**	27.5
**10**	**-0.11(-0.2 to 0.0)**	24.7	-0.11(-0.2 to 0.0)	45.1	-0.11(-0.3 to 0.0)	66.5	**-0.20(-0.3 to -0.1)**	55.4	**-0.22(-0.4 to -0.1)**	70.0	**-0.24(-0.4 to -0.1)**	83.3
**11**	-0.05(-0.2 to 0.1)	77.6	-0.06(-0.2 to 0.1)	83.4	-0.07(-0.3 to 0.1)	89.4	-0.06(-0.2 to 0.1)	83.1	-0.06(-0.2 to 0.1)	86.8	-0.06(-0.2 to 0.1)	91.2
**12**	-0.07(-0.2 to 0.1)	54.8	-0.06(-0.2 to 0.1)	61.8	-0.05(-0.2 to 0.1)	71.3	-0.06(-0.2 to 0.1)	73.9	-0.05(-0.2 to 0.1)	78.1	-0.04(-0.2 to 0.1)	83.5
**13**	-0.19(-0.4 to 0.1)	87.6	-0.21(-0.5 to 0.1)	89.1	-0.26(-0.5 to 0.0)	90.8	**-0.16(-0.2 to -0.1)**	40.9	**-0.16(-0.3 to -0.1)**	44.2	**-0.17(-0.3 to -0.1)**	51.3
**14-18**	-0.05(-0.2 to 0.1)	75.7	-0.05(-0.2 to 0.1)	75.7	-0.05(-0.2 to 0.1)	75.7	**-0.12(-0.2 to 0.0)**	72.9	**-0.12(-0.2 to 0.0)**	73.2	**-0.13(-0.2 to 0.0)**	73.9
**Overall**	**-0.07(-0.1 to 0.0)**	**81.3**	**-0.07(-0.1 to 0.0)**	**82.8**	**-0.06(-0.1 to 0.0)**	**85.3**	**-0.13(-0.2 to -0.1)**	**80.6**	**-0.14(-0.2 to -0.1)**	**82.6**	**-0.14(-0.2 to -0.1)**	**85.8**

Table S5. Sensitivity analysis showing estimated annual MVPA change (paired standardised mean difference) according to age in boys and girls using random effects (RE) model and quality effects (QE) model

Age (years)	Boys				Girls			
	RE Model		QE Model		RE Model		QE Model	
	SMD (95% CI)	p-value	SMD (95% CI)	p-value	SMD (95% CI)	p-value	SMD (95% CI)	p-value
3	-0.14(-0.8 to 0.5)	0.654	-0.18(-0.8 to 0.4)	0.575	-0.24(-0.8 to 0.4)	0.426	-0.25(-0.9 to 0.4)	0.412
4	**0.31(0.1 to 0.5)**	**0.009**	**0.31(0.1 to 0.5)**	**0.012**	**0.24(0.0 to 0.4)**	**0.023**	**0.24(0.0 to 0.4)**	**0.020**
5	0.24(-0.3 to 0.8)	0.422	0.23(-0.4 to 0.8)	0.462	0.26(-0.1 to 0.7)	0.205	0.30(-0.1 to 0.7)	0.162
6	0.10(-0.1 to 0.3)	0.367	0.09(-0.1 to 0.3)	0.442	**-0.19(-0.3 to 0.0)**	**0.021**	**-0.20(-0.4 to 0.0)**	**0.044**
7	0.10(-0.2 to 0.4)	0.530	0.03(-0.3 to 0.4)	0.869	-0.01(-0.3 to 0.3)	0.924	0.00(-0.4 to 0.4)	0.983
8	-0.09(-0.4 to 0.2)	0.499	0.01(-0.3 to 0.3)	0.965	-0.11(-0.4 to 0.2)	0.491	-0.10(-0.5 to 0.3)	0.580
9	**-0.20(-0.3 to -0.1)**	**<0.001**	**-0.21(-0.3 to -0.1)**	**<0.001**	**-0.28(-0.4 to -0.2)**	**<0.001**	**-0.28(-0.4 to -0.2)**	**<0.001**
10	-0.11(-0.2 to 0.0)	0.064	**-0.12(-0.2 to 0.0)**	**0.042**	**-0.20(-0.3 to -0.1)**	**0.001**	**-0.21(-0.4 to -0.1)**	**0.009**
11	-0.06(-0.2 to 0.1)	0.505	-0.08(-0.3 to 0.1)	0.379	-0.06(-0.2 to 0.1)	0.391	-0.05(-0.2 to 0.1)	0.584
12	-0.06(-0.2 to 0.1)	0.417	-0.09(-0.3 to 0.1)	0.276	-0.06(-0.2 to 0.1)	0.441	-0.08(-0.3 to 0.1)	0.393
13	-0.21(-0.5 to 0.1)	0.116	-0.09(-0.4 to 0.2)	0.557	**-0.16(-0.2 to -0.1)**	**0.001**	**-0.16(-0.3 to -0.1)**	**0.002**
14-18	-0.05(-0.2 to 0.1)	0.515	0.01(-0.1 to 0.2)	0.934	**-0.12(-0.2 to 0.0)**	**0.027**	-0.07(-0.2 to 0.0)	0.211
**Overall**	-0.03(-0.1 to 0.04)	0.407	-0.03(-0.1 to 0.0)	0.479	**-0.13(-0.2 to -0.1)**	**<0.001**	**-0.08(-0.1 to 0.0)**	**0.011**

Table S6. Sensitivity analysis showing estimated annual MVPA change (paired standardised mean difference) according to age in boys and girls using random effects (RE) model and quality effects (QE) model

	FE Model		RE Model	
	**SMD (95% CI)**	**p-value**	**SMD (95% CI)**	**p-value**
**Study design**				
Longitudinal studies	-0.07(-0.09 to -0.05)	<0.001	-0.06(-0.12 to -0.01)	0.030
Randomised Control Trials	-0.05(-0.08 to -0.01)	0.002	-0.07(-0.15 to 0.02)	0.159
Quasi Experimental	-0.17(-0.27 to -0.06)	<0.001	-0.22(-0.52 to 0.09)	0.125
**Epoch Length**				
<15s	-0.02(-0.05 to 0.00)	0.045	-0.03(-0.09 to 0.03)	0.363
30s	-0.03(-0.07 to 0.01)	0.207	-0.07(-0.18 to 0.04)	0.230
60s	-0.19(-0.22 to -0.15)	<0.001	-0.13(-0.23 to -0.02)	0.016
